# White matter tract correlations with spoken language in cerebrovascular disease

**DOI:** 10.1093/braincomms/fcaf145

**Published:** 2025-04-19

**Authors:** Dana N Broberg, Seyyed M H Haddad, Katharine Aveni, Alexander Havens, Paula M McLaughlin, Malcolm A Binns, Joseph B Orange, Stephen R Arnott, Courtney Berezuk, Leanne K Casaubon, Dar Dowlatshahi, Ayman Hassan, Nuwan D Nanayakkara, Alicia J Peltsch, Joel Ramirez, Gustavo Saposnik, Christopher J M Scott, Richard H Swartz, Sean Symons, Angela K Troyer, ONDRI Investigators, Agessandro Abrahao, Sabrina Adamo, Derek Beaton, Sandra Black, Alanna Black, Michael Borrie, Don Brien, Susan Bronskill, Dennis Bulman, Brian Coe, Ben Cornish, Sherif Defrawy, Jane Lawrence Dewar, Allison Ann Dilliott, Roger A Dixon, Sali Farhan, Frederico Faria, Elizabeth Finger, Corinne Fischer, Andrew Frank, Julia Fraser, Morris Freedman, Mahdi Ghani, Barry Greenberg, David Grimes, Wendy Hatch, Rob Hegele, Melissa Holmes, Chris Hudson, Mandar Jog, Peter Kleinstiver, Sanjeev Kumar, Donna Kwan, Tony Lang, Elena Leontieva, Brian Levine, Wendy Lou, Efrem Mandelcorn, Jennifer Mandzia, Ed Margolin, Connie Marras, Mario Masellis, Bill McIlroy, Manuel Montero-Odasso, Doug Munoz, David Munoz, Miracle Ozzoude, Stephen Pasternak, Bruce Pollock, Tarek Rajji, Natalie Rashkovan, John Robinson, Ekaterina Rogaeva, Demetrios Sahlas, Yanina Sarquis Adamson, Dallas Seitz, Christen Shoesmith, Alisia Southwell, Tom Steeves, Michael Strong, Stephen Strother, Sujeevini Sujanthan, Kelly Sunderland, Brian Tan, David Tang-Wai, Carmela Tartaglia, Faryan Tayyari, Athena Theyers, John Turnbull, Karen Van Ooteghem, John Woulfe, Modjeh Zamyadi, Lorne Zinman, Angela C Roberts, Robert Bartha

**Affiliations:** Department of Medical Biophysics, Schulich School of Medicine & Dentistry, Western University, London, ON N6A 3K7, Canada; Centre for Functional & Metabolic Mapping, Robarts Research Institute, Schulich School of Medicine & Dentistry, Western University, London, ON N6A 3K7, Canada; Centre for Functional & Metabolic Mapping, Robarts Research Institute, Schulich School of Medicine & Dentistry, Western University, London, ON N6A 3K7, Canada; Roxelyn & Richard Pepper Department of Communication Sciences & Disorders, Northwestern University, Evanston, IL 60201, USA; Department of Hearing and Speech Sciences, University of Maryland, College Park, MD 20742, USA; Roxelyn & Richard Pepper Department of Communication Sciences & Disorders, Northwestern University, Evanston, IL 60201, USA; School of Medicine, Case Western Reserve University, Cleveland, OH 44106, USA; Nova Scotia Health, Halifax, NS B3S 0H6, Canada; Departments of Medicine (Geriatrics) and Psychology & Neurosciences, Dalhousie University, Halifax, NS B3H 2Y9, Canada; Rotman Research Institute, Baycrest, Toronto, ON M6A 2E1, Canada; Dalla Lana School of Public Health, University of Toronto, Toronto, ON M5T 3M7, Canada; School of Communication Sciences & Disorders, Western University, London, ON N6G 1H1, Canada; Canadian Centre for Activity and Aging, Western University, London, ON N6G 1H1, Canada; Indoc Systems, Toronto, ON M5H 3W4, Canada; Harvard Medical School, Mass General Brigham, Boston, MA 02115, USA; Department of Medicine (Neurology), University of Toronto, Toronto, ON M5S 3H2, Canada; Department of Medicine (Neurology), University of Ottawa and Ottawa Hospital Research Institute, Ottawa, ON K1H 8M5, Canada; Northern Ontario School of Medicine University, Thunder Bay, ON P7B 5E1, Canada; Thunder Bay Regional Health Research Institute, Thunder Bay, ON P7B 7A5, Canada; Centre for Functional & Metabolic Mapping, Robarts Research Institute, Schulich School of Medicine & Dentistry, Western University, London, ON N6A 3K7, Canada; Smith Engineering, Queen’s University, Kingston, ON K7L 3N6, Canada; Dr. Sandra Black Centre for Brain Resilience and Recovery, Sunnybrook Research Institute, Toronto, ON M4N 3M5, Canada; Graduate Department of Psychological Clinical Science, University of Toronto Scarborough, Scarborough, ON M1C 1A4, Canada; Department of Medicine (Neurology), University of Toronto, Toronto, ON M5S 3H2, Canada; Sunnybrook Research Institute, Sunnybrook Health Sciences Centre, Toronto, ON M4N 3M5, Canada; Department of Medicine (Neurology), University of Toronto, Toronto, ON M5S 3H2, Canada; Hurvitz Brain Sciences Program, Sunnybrook Health Sciences Centre, Toronto, ON M4N 3M5, Canada; Department of Medical Imaging, Sunnybrook Health Sciences Centre, University of Toronto, Toronto, ON M4N 3M5, Canada; Neuropsychology & Cognitive Health Program, Baycrest, Toronto, ON M6A 2E1, Canada; Department of Psychology, University of Toronto, Toronto, ON M5S 3G3, Canada; School of Communication Sciences & Disorders, Western University, London, ON N6G 1H1, Canada; Department of Computer Science, Western University, London, ON N6A 5B7, Canada; Department of Medical Biophysics, Schulich School of Medicine & Dentistry, Western University, London, ON N6A 3K7, Canada; Centre for Functional & Metabolic Mapping, Robarts Research Institute, Schulich School of Medicine & Dentistry, Western University, London, ON N6A 3K7, Canada

**Keywords:** cerebrovascular disease, magnetic resonance imaging, diffusion tensor imaging, white matter, language

## Abstract

Assessment of spoken language is a promising marker for cognitive impairment in individuals with cerebrovascular disease. However, the underlying neurological basis for spoken language beyond single words and sentences remains poorly defined in this cohort, particularly with respect to white matter. This study aimed to examine and compare white matter hyperintensity volumes and diffusion tensor metrics in normal-appearing white matter (NAWM) as potential correlates of spoken language performance. Baseline imaging and spoken language data were obtained from the cerebrovascular disease cohort of the Ontario Neurodegenerative Disease Research Initiative (*n* = 127; age: 55–85 years). Most participants had subclinical or very mild strokes, with very little to no aphasia symptoms. Spoken language samples were analysed to compute 10 different measures related to syntax, productivity, lexical diversity, fluency, and information content. Structural and diffusion MRI data were analysed to segment white matter hyperintensities and tracts. Normalized white matter hyperintensity volume, as well as average fractional anisotropy and mean diffusivity in the normal-appearing portion of eight white matter tracts, were correlated with the 10 spoken language measures using canonical correlation analyses. White matter and spoken language variate scores for individual participants then were correlated separately in male (*n* = 86) and female (*n* = 41) participants to probe potential sex differences. Spoken language performance was significantly associated with the fractional anisotropy (*r_c_* = 0.51, *P*  *=* 0.041) and mean diffusivity (*r_c_* = 0.56, *P*  *=* 0.011) of NAWM, particularly in the left superior longitudinal fasciculus, but not with white matter hyperintensity volumes (*r_c_* = 0.41, *P*  *=* 0.80) in the same tracts. Measures related to syntax, fluency, and information content loaded most strongly in the spoken language variate. No significant sex differences were found in NAWM microstructure, and female and male participants exhibited similarly strong associations between spoken language and NAWM microstructure (fractional anisotropy: *z* = 1.44, *P* = 0.15; mean diffusivity: *z* = 1.03, *P* = 0.30). These results suggest that diffusion MRI in NAWM may be superior to white matter hyperintensity volumetrics when evaluating the role of white matter tract integrity on cognitive outcomes in people with relatively mild cerebrovascular pathology. These results also demonstrate that multi-domain spoken language analysis is sensitive to underlying white matter microstructure in participants with cerebrovascular disease without significant aphasia, supporting its value as a tool for assessing cognitive status.

## Teaser

Using a tract-based approach, Broberg *et al*. report that average diffusion tensor metrics within normal-appearing white matter correlate with spoken language in individuals with cerebrovascular disease, while white matter hyperintensity volumes do not. These results reinforce the value of diffusion imaging in linking white matter damage to cognitive outcomes.

## Graphical Abstract

**Graphical Abstract:**
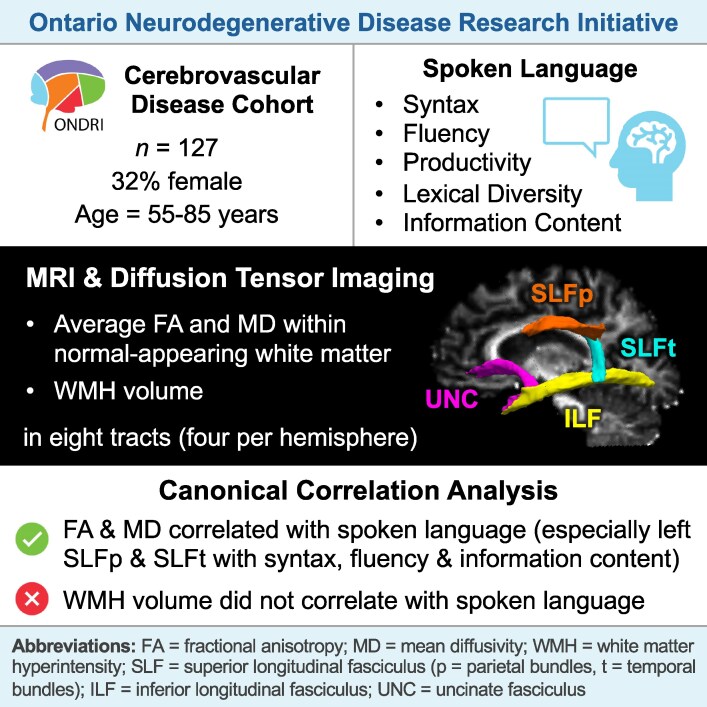

## Introduction

Cerebrovascular disease (CVD) is the second-leading cause of dementia after Alzheimer’s disease.^1^ CVD refers to a broad group of pathologies that, independently and in conjunction with other pathologies, increase dementia risk and accelerate cognitive decline.^1^ These include covert strokes with minimal to no observable cognitive or language symptoms, isolated or multiple infarcts, white matter lesions, lacunes, and small vessel disease—all of which can result in diminished cerebral blood flow leading to hypoxia, blood–brain barrier permeability, and the promotion of neurodegeneration and cognitive impairment.^2^ Notably, vascular cognitive impairment and vascular dementia can occur even in the absence of a major vascular event (e.g. stroke).^3^ There are several modifiable risk factors for vascular dementia, including hypertension, hypercholesterolaemia, smoking, obesity, and diabetes,^4^ making early detection critical for people at risk for vascular dementia using sensitive and specific measures of cognitive function.

Spoken language has demonstrated sensitivity for detecting vascular cognitive impairment.^5^ Recently, a study examining the CVD cohort of the Ontario Neurodegenerative Disease Research Initiative (ONDRI)^6^ used a multi-domain spoken language analysis (which included measures of syntax, productivity, lexical diversity, fluency, and information content) to determine the spoken language signature of cognitive impairment in CVD. Roberts *et al.*^5^ found that a combination of 10 spoken language measures correctly classified 78% of the CVD participants as cognitively impaired or cognitively normal for their age group, with sensitivity of 77% and specificity of 80%—accuracy similar to cognitive screening tools used clinically.

Previous studies of people living with dementia have drawn links between spoken language and various neurological changes (e.g. grey matter atrophy, white matter diffusion),^7–11^ helping to validate that spoken language assessments relate to underlying neuropathology. However, the neurological basis for impaired spoken language in dementia beyond primary progressive aphasia and in vascular dementia, specifically, is less clear.^5^ Understanding vascular contributions to cognitive and language impairments in dementia is important because mixed pathologies are common, with upwards of 61% of cases with frontotemporal dementia and 82% of cases with Alzheimer’s disease showing evidence of co-morbid cerebrovascular disease.^12^

Previous neurolinguistic studies have focused largely on cortical structures and grey matter. As a result, less is known about white matter tract contributions to spoken language in neurodegenerative diseases. However, white matter tracts play a key role in cognitive dysfunction,^1^ and network-based approaches are critical for resolving symptoms in cases where lesions are distant from cortical regions dedicated to that cognitive function.^13^ Additionally, white matter tracts are particularly important for understanding vascular contributions to cognitive impairment, given that white matter contains 70–75% less vasculature,^14^ has significantly lower cerebrovascular reactivity^15^ than grey matter, and therefore may be more vulnerable than grey matter when cerebral blood flow is disrupted.^16^

Many—but not all—people with CVD have visible white matter lesions, such as infarcts, lacunes, and white matter hyperintensities (WMH), all of which can be associated with cognitive decline and dementia^17^ and could also impact spoken language performance. However, visible lesions may represent only a small component of a greater underlying phenomenon. While WMH have been linked to ischaemic damage, hypoperfusion, demyelination, axonal loss, reduced glial density,^18^ venous collagenosis,^19^ and endothelial or immune activation,^18^ similar pathological changes can also be observed in normal-appearing white matter (NAWM).^20^ NAWM is white matter that contains no visible lesions and directly contributes to the structural connectivity between various brain regions and, consequently, to cognitive performance. Probing NAWM microstructure may provide important insight into early-stage damage within white matter tracts that is not captured by visible lesions, and could help overcome limitations of using indirect measures of white matter integrity based on lesions to predict language ability.^21^

Diffusion tensor imaging (DTI) provides a sensitive, quantitative assessment of white matter tissue microstructure. Specifically, DTI can quantify parameters that describe water diffusion characteristics, such as fractional anisotropy (FA) and mean diffusivity (MD), which are known to change in neurodegeneration.^22^ Degeneration of white matter microstructure is often characterized by reduced FA and increased MD.^23^ Previous studies of the neural mechanisms of spoken language examined white matter using DTI, but none included participants with cognitive impairment due to cerebrovascular disease in the absence of stroke-related aphasia or motor speech disorders.^7,8,11,24–29^ However, in a study of subcortical ischaemic vascular dementia, Liao *et al.*^30^ found that FA and MD of NAWM were significantly associated with Montreal Cognitive Assessment^31^ scores, whereas FA and MD of whole-brain white matter, as well as white matter lesion volume, were not. The authors concluded that microstructural damage to NAWM may play an important role in vascular dementia pathogenesis. Given these findings, we were interested in using DTI to determine whether NAWM microstructure is also related to spoken language performance and how WMH and NAWM might differ as correlates of spoken language performance.

Therefore, the current study aimed to examine whether WMH volume and DTI metrics of tissue microstructure in NAWM correlate with measures of spoken language in people with cerebrovascular disease at high risk for dementia but without significant stroke-related aphasia or motor speech symptoms. We focused our analyses on 10 spoken language measures previously found to be indicative of cognitive impairment in this cohort.^5^ We considered four white matter tracts in each hemisphere that are known to be involved in spoken language based on previous literature on neural correlates of spoken language and the dual-stream model of language processing^32–34^: the superior longitudinal fasciculus parietal bundle (SLFp) and superior longitudinal fasciculus-temporal bundle (SLFt) contributing to the dorsal pathway (the SLFp and SLFt correspond most closely to the SLF III and arcuate fasciculus, respectively^35^), and the inferior longitudinal fasciculus (ILF) and uncinate fasciculus (UNC) contributing to the ventral pathway. The primary objective of this study was to understand the neural contributions to spoken language impairments in CVD by determining whether WMH volume and diffusion metrics in the NAWM of the white matter tracts were associated with multi-domain spoken language performance. We hypothesized that increased WMH volume, decreased FA, and increased MD in the white matter tracts would correspond to worse performance on spoken language measures.

## Materials and methods

### Participants

The ONDRI study is a multi-site longitudinal study aimed at carefully characterizing five neurodegenerative diseases (Alzheimer’s disease, Parkinson’s disease, frontotemporal dementia, amyotrophic lateral sclerosis, and CVD) across multiple domains of assessments, including neuroimaging, cognition, language, gait, genomics, retinal imaging, and eye-tracking. Previous publications have detailed the ONDRI study, including the inclusion and exclusion criteria for each cohort.^6,36^ The ONDRI research protocol was approved by the Research Ethics Boards at each of the 14 participating institutions.^6^

The current study used clinical assessment, neuroimaging, cognition, and spoken language data from the CVD cohort at baseline. Participants in the CVD cohort were recruited by clinical stroke neurologists at academic health centers across Ontario, Canada. Participants with CVD were eligible for the study if they had experienced a mild to moderate ischaemic stroke or transient ischaemic attack confirmed by imaging at least 3 months before enrolment or had severe white matter disease with subcortical lacunar infarcts observed on MRI. It should be noted that several participants in the CVD group did not have visible lesions at the time of enrolment even though they had a confirmed history of having a stroke. Individuals with non-vascular aetiologies, large cortical strokes, or severe cognitive impairment, aphasia, or dysarthria were excluded. The CVD cohort was sampled intentionally such that approximately half the cohort had detectable cognitive impairment on screening with the Montreal Cognitive Assessment^31^ (scores of 18–25 out of a maximum of 30), with the remaining participants having scores between 26 and 30. This intentional sampling approach was designed to facilitate monitoring the evolution of cognitive changes over time (worsening in those with existing cognitive impairment and onset of cognitive decline in those without evidence of impairment at baseline based on neuropsychological assessment^6,36^) within this longitudinal study. Participants self-reported English as their primary language, self-rated their speaking and understanding of English with a score of at least 7 (out of a maximum of 10) on the Language Experience and Proficiency Questionnaire,^37^ and had at least 8 years of formal education. The ONDRI study enrolled 161 participants with CVD at baseline. Of these, 131 participants had usable data for both the spoken language and imaging analyses, which are detailed below. Demographic and clinical characteristics of the participants included in this study are provided in Table 1. Based on NIH Stroke Scale ratings made by stroke clinicians, 98% of participants had a rating of no aphasia, with the remainder having mild aphasia, and 97% of participants were rated as having ‘normal’ speech (no dysarthria), with the remainder having mild symptoms. Additional clinical characteristics are provided in Supplementary Table 1. Lacunes and stroke lesions were present in 56% and 92% of participants at the time of study imaging visits, respectively. The prevalence of lacunes and stroke lesions within specific brain regions is described in Supplementary Table 2. Neuropsychological assessment results are described in Roberts *et al*.^5^

**Table 1:** Participant demographics and clinical characteristics

	Females (*n* = 44)		Males (*n* = 87)	
Continuous variable	Median (IQR)	Range	Median (IQR)	Range
OR	*n*	%	*n*	%
Categorical/ordinal variable				
**Demographics**				
Age	68.7 (12.9)	55.0–84.5	69.5 (10.1)	55.5–85.4
Education (years)	14 (4)	11–20	14 (4)	8–20
**Ethnicity**				
White	34	77.3%	74	85.1%
Asian	3	6.8%	8	9.2%
Black	3	6.8%	3	3.4%
Other	3	6.8%	2	2.3%
Multiple	1	2.3%	0	0.0%
**Handedness**				
Right	39	88.6%	78	89.7%
Left	4	9.1%	8	9.2%
Ambidextrous	1	2.3%	1	1.1%
**mRS total score**				
0	13	29.5%	28	32.2%
1	17	38.6%	38	43.7%
2	12	27.3%	19	21.8%
3	2	4.5%	1	1.1%
4	0	0.0%	1	1.1%
**MoCA total score**	26.0 (6.0)	18–30	26.0 (5.0)	18–30
**Stroke history**				
Years since stroke^a^	1.3 (3.6)	0.2–12.5	0.7 (1.3)	0.2–34.5
Presence of subclinical stroke				
Yes	6	13.6%	14	16.1%
No	36	81.8%	71	81.6%
Unknown	2	4.5%	2	2.3%
**MRI volumetrics**				
Total intracranial volume (cm^3^)	1085 (146)	918–1397	1278 (155)	1034–1578
Normal-appearing white matter (cm^3^)	347.8 (68.8)	236.7–443.0	406.3 (63.0)	275.3–528.3
White matter hyperintensities (cm^3^)	7.5 (17.7)	0.6–55.2	4.1 (9.1)	0.3–84.1
Lacunar volume (mm^3^)	121 (409)	0–2407	95 (290)	0–6220
Stroke lesion volume (cm^3^)	0.3 (4.6)	0.0–68.2	0.2 (6.6)	0.0–43.7

IQR = interquartile range; mRS = modified Rankin Scale^38^; MoCA = Montreal Cognitive Assessment^31^; MRI = magnetic resonance imaging.

^a^The date of stroke for 15 participants was unknown (8 of these participants had confirmed subclinical strokes).

### Spoken language

The spoken language data included in the current study were processed and analysed as part of ongoing ONDRI research conducted by researchers in author A.R.’s laboratory. The study protocol, spoken language results, annotation, analysis, and reliability assessment procedures and results for the ONDRI CVD cohort are reported in detail in Roberts *et al*.^5^ Briefly, spoken language samples at baseline were elicited using the ‘Argument’ picture sequence, a standardized spoken language elicitation stimulus from the Nicholas and Brookshire stimuli battery.^39^ Participants were instructed to generate a narrative with these instructions^40^: ‘I am going to ask you to tell a story. Look at this series of pictures to familiarize yourself with the story.’ Participants were provided 60–90 s to preview the pictures before examiners prompted participants with: ‘Now use these pictures to tell me a story in as much detail as you can.’ Examiners were permitted to repeat the instructions once. There was no maximum time limit for the task.

Spoken language samples were recorded digitally with an AKG 520C, head-worn, condenser microphone connected to a PC laptop via a Scarlett 2i2 USB preamplifier. Using previously published methods, spoken language samples were transcribed orthographically, segmented into utterances using a communication unit convention^41^ that defines an utterance as the independent (main) clause and its modifiers, and annotated for the spoken language behaviours of interest by trained research staff.^5,42^ The 10 spoken language variables included in the current analysis (Table 2) were those identified by Roberts *et al*.^5^ as significantly discriminating participants with cognitive impairment from those without cognitive impairment, based on a comprehensive neuropsychological assessment battery, within the CVD cohort. Inter- and intra-rater reliability procedures for the spoken language annotation and other data quality measures for the CVD cohort are detailed in Roberts *et al*.^5^ Inter-rater reliability across variables ranged from an intra-class correlation coefficient of 0.850 to 0.999. Intra-rater reliability ranged from an intra-class correlation coefficient of 0.924 to 1.00. Reliability was excellent for all spoken language variables. Speech intelligibility was high, with 99.6% of words being able to be correctly transcribed and few unintelligible words across samples.

**Table 2:** Spoken language measure definitions^5,42,43^

Measure	Definition
**Syntax**	
Mean length of utterance (MLU)	Mean length of utterance in words for intelligible, complete, verbal, and task-relevant utterances^44^
Subordination index (SI)	Subordination index composite score; ratio of total number of clauses + predicate clauses:total number of utterances.^45^ Phrase-level dysfluencies (multi-word revised phrases or utterances) were not included in these analyses.
Clauses per utterance (Clauses/Ut)	Ratio of total number of clauses:total number of utterances (similar to SI, except that infinitive clauses were counted, and clauses with implicit subjects were counted so long as the main clause had a subject present).^5^ Phrase-level dysfluencies (multi-word phrases or utterances) were not included in these analyses.
**Productivity**	
Total words	Number of full, intelligible words in context; nonword fillers were not counted; contractions and common simplifications counted as separate words; proper names, titles, and compound words were counted as separate words^39^
Words per minute (WPM)	Total words ÷ participant speaking time in minutes^44^
**Lexical diversity**	
Moving-average type-token ratio (MATTR)	SALT-generated moving-average ratio of different words:total words with a window size of 23 words representing the shortest language sample in the database.^46,47^
**Fluency**	
% Maze words	Maze words (i.e. filled pauses, false starts, reformulations, and interjections) ÷ [maze words + non-maze words]^5^
Word-level dysfluencies per utterance (Wd dys/Ut)	(Total number of word, syllable, and sound repetitions + total number of initial, middle, and final sound prolongations)^48^ ÷ total utterances
**Information content**	
% Main events	Proportion of correct narrative main events^49^
Correct information units per minute (CIUs/min)	Total number of CIUs (i.e. number of words intelligible in context and accurate, relevant, and informative about the picture content) ÷ total participant speaking time in minutes^39^

An utterance was defined as a main clause and its accompanying dependent clauses. However, conjoined main clauses were segmented into separate utterances, even when the subject of the second clause was implicit. Correct information units (CIUs) were defined as words intelligible in context and accurate, relevant, and informative about the picture content.

### Neuroimaging data acquisition

MRI scans were fully evaluated for incidental findings by a neuroradiologist (S.S.) and assessed to ensure high imaging quality by a medical biophysics scientist (R.B.). The MRI acquisition protocol for the ONDRI study has been described previously^6^ and was standardized, consistent with the Canadian Dementia Imaging Protocol,^50^ to allow the pooling of data from 11 different 3.0 T scanners [General Electric (Chicago, IL, USA), Siemens (Erlangen, Germany) and Philips (Amsterdam, Netherlands)] across Ontario, Canada. The protocol included the following sequences: T_1_-weighted MRI (1 mm isotropic), interleaved proton density/T_2_-weighted MRI (0.94 × 0.94 × 3 mm^3^), fluid-attenuated inversion recovery MRI, T_2_*-weighted MRI, resting-state functional MRI, and diffusion MRI (2 mm isotropic, 30–32 directions, *b*-value = 1000 s/mm). As part of the DTI protocol, at least one image with no diffusion weighting (b0 volume) was also acquired. The use of 30–32 directions without acceleration allowed the use of tensor modelling within the constraints of total scan time and scanner capabilities. Strict quality control measures for the DTI data are described in Haddad *et al.*^51^

#### Segmentation of cerebral tissues and vascular lesions

Brain tissue types, including stroke volumes, lacunes, WMH, and NAWM, were segmented using the ONDRI structural MR image processing pipeline referred to as Semi-Automatic Brain Region Extraction-Lesion Explorer (SABRE-LE).^52^ The interleaved proton density/T_2_-weighted and fluid-attenuated inversion recovery images were co-registered to the T_1_-weighted images, and proton density/T_2_-based masks were produced. These masks were then used to segment the T_1_-weighted images using a multi-feature histogram method^53^ into grey matter, white matter, and CSF. WMH and lacunes were automatically identified using Fuzzy Lesion Extractor^54^ and Lesion Explorer^55,56^ and manually edited. Cortical strokes were segmented manually. NAWM was defined by excluding WMH and lacunes from white matter regions. MRI-derived volumes of total intracranial volume, NAWM, WMH, lacunes, and stroke lesions are described in Table 1.

### Automated DTI data analysis and tractography

DTI analysis and tractography for the segmentation of 18 major white matter tracts were accomplished using TRACULA (TRActs Constrained by UnderLying Anatomy, https://surfer.nmr.mgh.harvard.edu/fswiki/Tracula)^35^ in FreeSurfer (version 6.0.0), a software that is freely available and well-documented online.^57–59^ TRACULA uses prior information about white matter pathway trajectories relative to surrounding subcortical and cortical structures obtained from a training set of manually labelled tracts to produce tractography streamlines. While the training set is from cognitively normal subjects, TRACULA has been successfully applied in a variety of clinical populations,^60^ including neurodegenerative disease cohorts with lesions.^61^

First, cortical and subcortical segmentations were performed on the T_1_-weighted images using FreeSurfer’s ‘recon-all,’ which delineates specific brain regions to inform the fibre tracking procedure in TRACULA. DTI data were preprocessed to correct for eddy currents and motion and then registered to the corresponding T_1_-weighted image. Both the T_1_-weighted image and DTI data were then registered to the MNI152 template. Diffusion tensor fitting was performed to obtain the conventional DTI maps, including FA and MD maps. Finally, through automated global probabilistic tractography with anatomical neighbourhood priors in TRACULA, 18 major white matter tracts were segmented in each subject.^35^ More specifically, TRACULA uses the tract endpoints from the training set in combination with each subject’s cortical and subcortical anatomy to establish and constrain probabilistic streamlines. Then, a Markov Chain Monte Carlo algorithm iteratively perturbs control points to dictate the curvature of the tract and update its posterior probability distribution. This approach, which does not assume exact tract locations or shapes, enables reliable tract reconstruction across individuals with varying underlying anatomy. In the end, TRACULA generates probabilistic spatial distributions for 18 white matter tracts, including the SLFp and SLFt as dorsal language pathways (which correspond most closely to the SLF III and arcuate fasciculus, respectively^35^), and the ILF and UNC as ventral language pathways. These probabilistic distributions were thresholded at 50% of their maximum probability to create the initial tract segmentation masks.

### Removal of lesions from white matter tract segmentation masks

The white matter tract segmentation masks generated by TRACULA in native T_1_-weighted image space include WMH and other white matter anomalies such as stroke lesions and lacunar infarcts. Using the SABRE-LE lesion masks generated in the same space as described above, all these anomalies were excluded from the white matter segmentation masks using a custom-developed MATLAB code (MATLAB R2019, Natick, MA, USA: The MathWorks Inc., 2019), which calls FSL masking instructions (https://fsl.fmrib.ox.ac.uk/fsl/fslwiki/Fslutils). Using this strategy, the only tissue remaining in the white matter segmentation masks produced by TRACULA was NAWM, while all other tissue/lesion types were excluded. Then, using these tract-based NAWM masks and DTI maps obtained from TRACULA, the average FA and MD in the NAWM tissue of each of the white matter tracts were quantified as a measure of the overall integrity of the NAWM part of those tracts. Note that while axial diffusivity and radial diffusivity were also calculated, previous work showed that MD highly correlates with both axial diffusivity (*r* = 0.990, *P* < 0.001) and radial diffusivity (*r* = 0.998, *P* < 0.001) in the ONDRI CVD cohort,^51^ so these measures were not included in the current statistical analyses. Additionally, the SABRE-LE lesion masks were used to create masks of the WMH portion of each tract. The volumes of these masks were used to calculate the normalized WMH volume in each tract as (WMH volume/Total intracranial volume).

### Statistical analysis

Statistical analyses were performed using IBM SPSS Statistics Version 28.0 (IBM Corp., Armonk, NY, USA), GraphPad Prism Version 9.4 (GraphPad Software, San Diego, CA, USA), and Microsoft Excel 16.6 (Microsoft Corp., Redmond, WA, USA). Differences in the DTI and spoken language variables between females and males were evaluated using four multivariable general linear models—one for each imaging variable (FA, MD, WMH) in each tract and one for the spoken language variables—with sex as a fixed effect. If a multivariable analysis was statistically significant, univariate analyses were subsequently performed on each variable to evaluate the effect of sex with the false discovery rate controlled using the Benjamini–Hochberg procedure with *q* = 0.05.^62^

Canonical correlation analysis (CCA) was used to model the association between DTI and spoken language. Specifically, CCA was performed separately for WMH volume, FA, and MD. Multivariate outliers were identified by calculating the Mahalanobis distance^63^ for each subject based on the 34 variables included in the CCAs (WMH volume, FA, and MD in the NAWM of the eight tracts, plus the 10 spoken language measures). The Mahalanobis distances were then evaluated using a *χ*^2^ distribution with *df* = 34, with *P*  *<* 0.001 as the statistical threshold recommended by Tabachnick and Fidell.^64^

Each CCA included eight white matter tracts (Variate 1; the left and right ILF, SLFp, SLFt and UNC) and 10 spoken language measures (Variate 2) and provided eight different sets of variates (also referred to as roots), each with its own canonical correlation coefficient and eigenvalue, which were evaluated for statistical significance using Wilks’ lambda distribution. CCA also provided canonical loadings, representing a variable’s correlation with its variate (e.g. total words with Variate 2). Canonical loadings were used to interpret the importance of each variable in the association between spoken language and the white matter tracts. CCA was repeated with the outliers removed to evaluate the influence of the outlying participants on the CCA results. Bivariate Pearson cross-correlations were also computed to support CCA interpretation. False discovery rate for these correlations was controlled using the Benjamini–Hochberg procedure with *q* = 0.05.^62^

A follow-up analysis investigated whether sex affected the association between white matter tract microstructure and spoken language performance. For each participant, we calculated scores for the white matter variate and the spoken language variate from the statistically significant CCAs based on the unstandardized weights of each CCA variable. The white matter variate scores and spoken language variate scores of each participant were then correlated to one another within each sex to evaluate the effect of sex on the strength of the association between spoken language and DTI. To mitigate the influence of the different sample sizes of each sex, Pearson *r* values were transformed to Fisher *z*-scores for comparison. Statistical significance for all analyses was set at a threshold of *α* = 0.05 (except for the multivariate outlier analysis using Mahalanobis distance as described above).

## Results

### White matter and spoken language variables

**Figure 1:**
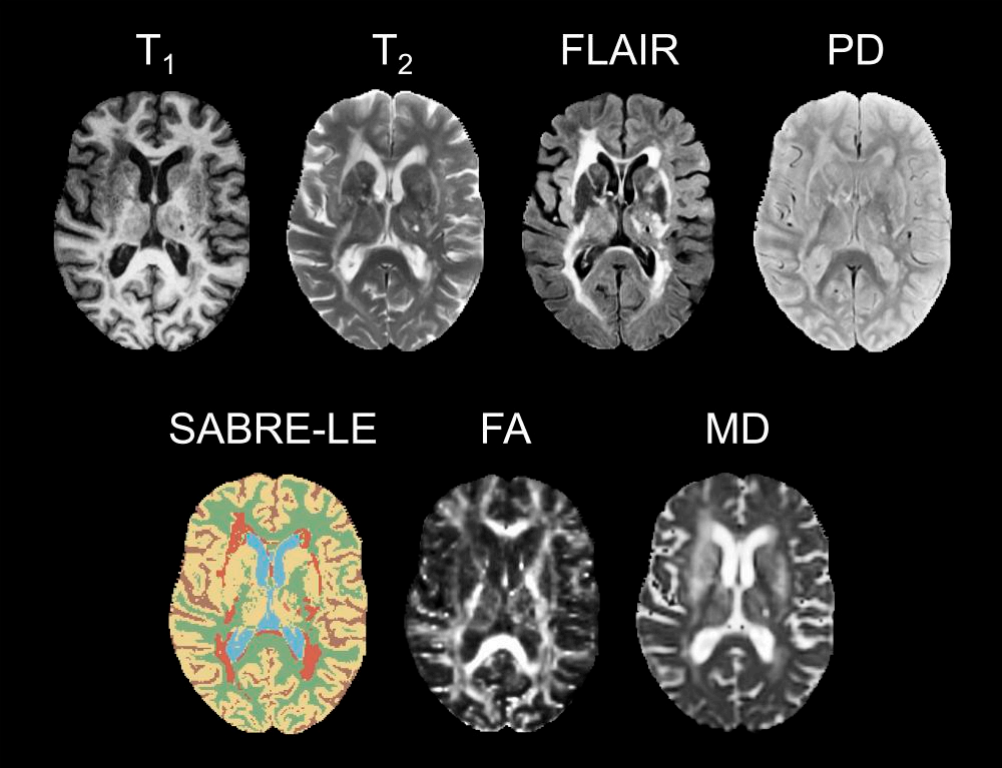
**Sample MR images**. Examples of the various types of MR images acquired (T_1_, T_2_, FLAIR, PD) or generated (SABRE-LE, FA, MD) in the current study are shown for a single participant with cerebrovascular disease. The SABRE-LE image shows the segmentation of normal-appearing grey matter (beige), normal-appearing white matter (green), white matter hyperintensities (orange), periventricular lacune (lime green), ventricular CSF (blue), and sulcal CSF (brown). FLAIR = fluid-attenuated inversion recovery; PD = proton density; SABRE-LE = Semi-Automatic Brain Region Extraction-Lesion Explorer^52^; FA = fractional anisotropy; MD = mean diffusivity.

**Figure 2:**
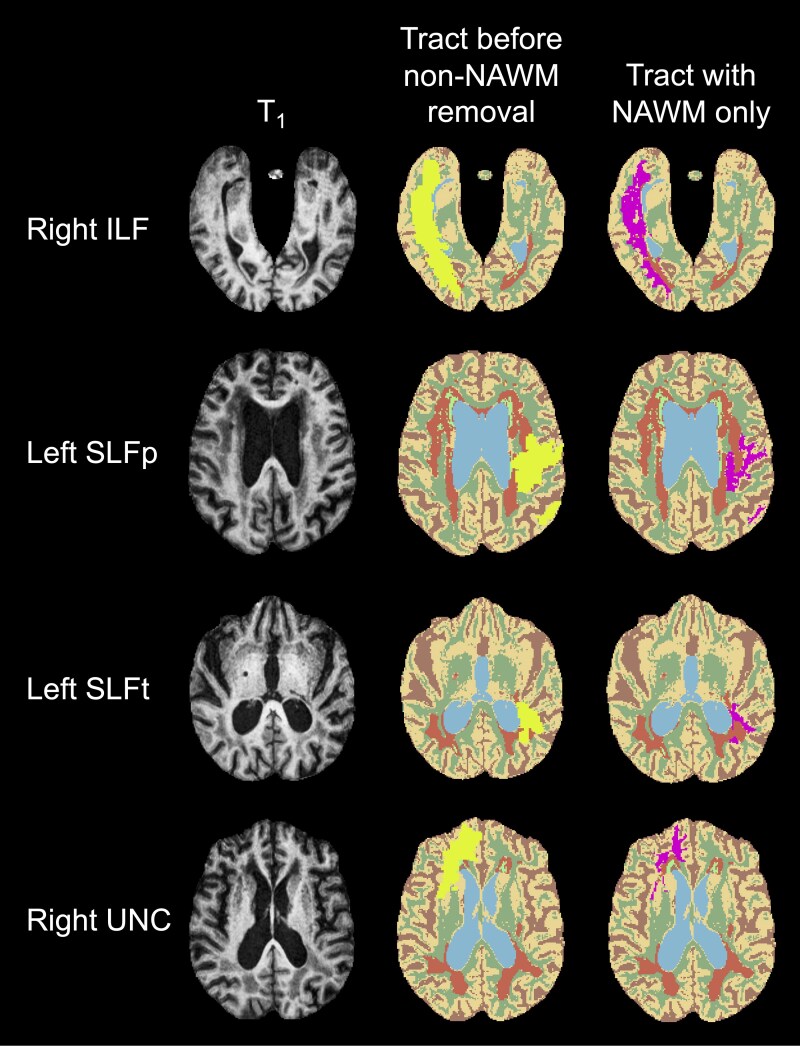
**Extraction of normal-appearing white matter (NAWM) in selected white matter pathways implicated in spoken language.** Shown are segmentations of four white matter tracts (ILF, SLFp, SLFt, and UNC) obtained using TRACULA before (yellow) and after (magenta) removal of white matter lesions and non-NAWM, along with corresponding T_1_-weighted anatomical images. The tract segmentations are superimposed on the Semi-Automatic Brain Region Extraction-Lesion Explorer (SABRE-LE)^52^ images (beige = normal-appearing grey matter; green = NAWM; orange = white matter hyperintensities; lime green = periventricular lacune; blue = ventricular CSF; brown = sulcal CSF). For each tract, mean diffusion tensor imaging (DTI) metrics were calculated in NAWM only. Note that white matter pathway streamlines are visualized in Supplementary Fig. 1. ILF = inferior longitudinal fasciculus; SLFp = superior longitudinal fasciculus—parietal bundle; SLFt = superior longitudinal fasciculus-temporal bundle; UNC = uncinate fasciculus.

Examples of the MR images acquired and generated in the current study are shown in Fig. 1, including the SABRE-LE images and FA and MD maps. Segmentations of the white matter tracts before and after the removal of WMH and non-NAWM are shown in Fig. 2, while 3D tractograms before thresholding and non-NAWM removal are shown in Supplementary Fig. 1. Note that, on average, the SLFp and SLFt overlapped 8.0% and 4.5% in the left and right hemispheres, respectively.

Descriptive statistics of the white matter tract and spoken language variables for each sex are provided in Tables 3 and 4, respectively. The multivariate general linear models revealed that sex did not have a significant effect on FA (F_8,122_ = 1.15, *P*  *=* 0.34, $\eta_{p}^{\;2}$ = 0.070) or MD (F_8,122_ = 0.56, *P*  *=* 0.81, $\eta_{p}^{\;2}$ = 0.035). However, sex did significantly affect spoken (F_10,120_ = 2.33, *P*  *=* 0.015, $\eta_{p}^{\;2}$ = 0.16) as well as normalized WMH volume (F_8,122_ = 5.41, *P*  *<* 0.001, $\eta_{p}^{\;2}$ = 0.26). Subsequent univariate analyses of the 10 spoken language variables revealed that none of the language variables differed significantly between males and females after controlling for false discovery rate. Univariate analyses of normalized WMH volume in each of the tracts revealed that females had more WMH in the left UNC [females: mean (SD) = 0.1 (0.4), males: mean (SD) = 0.0 (0.1), *P* = 0.015, $\eta_{p}^{\;2}$ = 0.045], right SLFp [females: mean (SD) = 1.2 (1.9), males: mean (SD) = 0.2 (0.5), *P* < 0.001, $\eta_{p}^{\;2}$ = 0.15], and right SLFt [females: mean (SD) = 1.6 (2.7), males: mean (SD) = 0.1 (0.3), *P* < 0.001, $\eta_{p}^{\;2}$ = 0.18] compared to men, after controlling for false discovery rate.

**Table 3:** Descriptive statistics of white matter variables (*n* = 131)

	Females (*n* = 44)			Males (*n* = 87)		
		95% confidence interval			95% confidence interval	
	Mean (SD)	Lower	Upper	Mean (SD)	Lower	Upper
**FA**						
Left ILF	0.50 (0.05)	0.49	0.52	0.51 (0.06)	0.50	0.53
Left SLFp	0.47 (0.06)	0.45	0.49	0.48 (0.05)	0.47	0.49
Left SLFt	0.49 (0.06)	0.47	0.51	0.49 (0.05)	0.48	0.50
Left UNC	0.49 (0.07)	0.47	0.51	0.49 (0.07)	0.48	0.51
Right ILF	0.50 (0.05)	0.49	0.52	0.50 (0.05)	0.49	0.52
Right SLFp	0.46 (0.05)	0.45	0.48	0.48 (0.05)	0.47	0.49
Right SLFt	0.49 (0.05)	0.48	0.51	0.49 (0.05)	0.48	0.50
Right UNC	0.46 (0.05)	0.45	0.48	0.47 (0.05)	0.46	0.48
**MD**						
Left ILF	0.84 (0.06)	0.82	0.86	0.84 (0.05)	0.83	0.85
Left SLFp	0.78 (0.07)	0.76	0.80	0.77 (0.05)	0.76	0.78
Left SLFt	0.79 (0.06)	0.77	0.80	0.78 (0.04)	0.77	0.79
Left UNC	0.81 (0.06)	0.79	0.82	0.81 (0.06)	0.80	0.82
Right ILF	0.84 (0.07)	0.82	0.86	0.84 (0.07)	0.83	0.86
Right SLFp	0.77 (0.06)	0.75	0.79	0.77 (0.06)	0.76	0.78
Right SLFt	0.78 (0.06)	0.77	0.80	0.78 (0.05)	0.77	0.79
Right UNC	0.82 (0.07)	0.80	0.84	0.82 (0.06)	0.81	0.84
**WMH vol (×10^5^)**						
Left ILF	0.1 (0.9)	0.0	0.4	0.1 (0.6)	0.0	0.2
Left SLFp	0.7 (2.5)	0.0	1.5	0.3 (1.6)	0.0	0.6
Left SLFt	0.8 (2.1)	0.2	1.4	0.2 (0.7)	0.1	0.4
Left UNC^a^	0.1 (0.4)	0.0	0.2	0.0 (0.1)	0.0	0.0
Right ILF	0.2 (0.9)	0.0	0.5	0.1 (0.8)	0.0	0.3
Right SLFp^a^	1.2 (1.9)	0.6	1.8	0.2 (0.5)	0.1	0.2
Right SLFt^a^	1.6 (2.7)	0.8	2.5	0.1 (0.3)	0.0	0.1
Right UNC	0.0 (0.4)	0.0	0.2	0.0 (0.2)	0.0	0.1

Average fractional anisotropy (FA) and mean diffusivity (MD) values within normal-appearing white matter, along with normalized white matter hyperintensity (WMH) volumes, are reported for each white matter tract. ILF = inferior longitudinal fasciculus; SLFp = superior longitudinal fasciculus (parietal bundles); SLFt = superior longitudinal fasciculus (temporal bundles); UNC = uncinate fasciculus.

^a^Significant difference between females and males after controlling for false discovery rate.

**Table 4:** Descriptive statistics of spoken language variables (*n* = 131)

	Females (*n* = 44)			Males (*n* = 87)		
Variable	Mean (SD)	95% confidence interval		Mean (SD)	95% confidence interval	
		Lower	Upper		Lower	Upper
MLU	6.68 (1.85)	6.12	7.25	6.90 (1.42)	6.60	7.20
SI	1.10 (0.27)	1.02	1.18	1.04 (0.20)	1.00	1.08
Clauses/Ut	1.39 (0.32)	1.29	1.49	1.38 (0.24)	1.33	1.43
Total words	133.1 (58.6)	115.3	150.9	140.7 (64.2)	127.0	154.3
WPM	143.3 (33.9)	133.0	153.6	148.6 (35.9)	140.9	156.2
MATTR	0.82 (0.04)	0.80	0.83	0.84 (0.04)	0.83	0.84
% Maze words	4.03 (3.58)	2.94	5.12	5.77 (5.73)	4.55	6.99
Wd dys/Ut	0.15 (0.20)	0.09	0.21	0.11 (0.15)	0.08	0.15
% Main events	62.0 (19.7)	56.0	68.0	55.8 (23.3)	50.9	60.8
CIUs/min	101.5 (31.6)	91.9	111.1	102.1 (28.5)	96.0	108.2

MLU = mean length of utterance (in words); SI = subordination index; Clauses/Ut = mean number of clauses per utterance; WPM = words per minute; MATTR = moving-average type-token ratio; % Maze words = percentage of maze words; Wd dys/Ut = word-level dysfluencies per utterance; % Main events = proportion of main events; CIUs/min = correct information units per minute. No variables differed significantly between males and females after controlling for false discovery rate. Spoken language variables are defined in Roberts *et al.*^5^ and Table 2.

### Canonical correlation analysis of white matter with spoken language

CCA was performed to determine whether WMH volume, FA, or MD were associated with spoken language measures. In these analyses, none of the canonical variates for FA and WMH volume were statistically significant (FA: *n* = 131, *r_c_* = 0.51, *P*  *=* 0.23; WMH: *n* = 131, *r_c_* = 0.40, *P*  *=* 0.76), while the CCA for MD had one set of canonical variates that was (*n* = 131, *r_c_* = 0.55, *P*  *=* 0.016).

**Figure 3:**
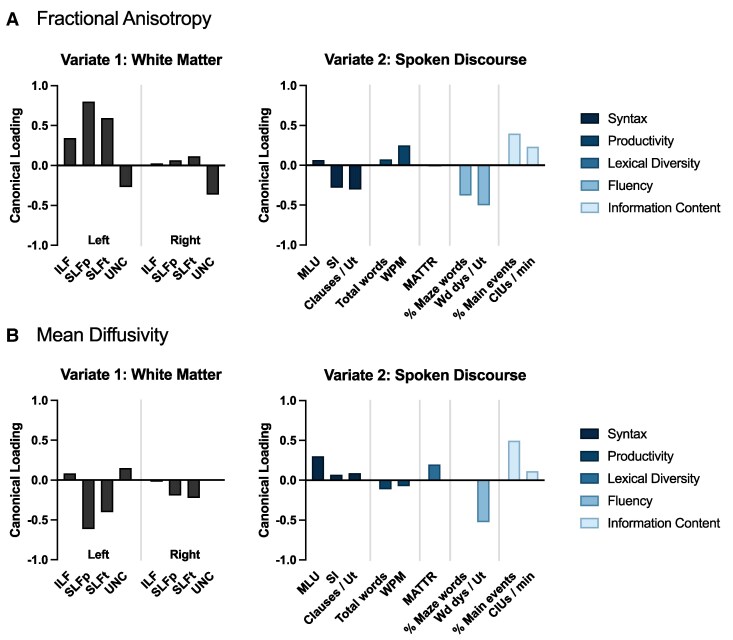
**Canonical correlation loadings of diffusion tensor imaging and spoken language measures in individuals with cerebrovascular disease.** Canonical correlations examined the association between diffusion tensor imaging (DTI) metrics in both hemispheres of the brain and spoken language performance. The canonical loadings of each DTI and spoken language variable onto their respective variates are shown for (**A**) fractional anisotropy (*n* = 127, *r_c_* = 0.509, *P*  *=* 0.041) and (**B**) mean diffusivity (*n* = 127, *r_c_* = 0.557, *P*  *=* 0.011). Spoken language variables can be categorized into several performance domains, as shown in the legends. Tracts with larger magnitude canonical loadings are interpreted as having stronger associations with spoken language measures that also had larger magnitude canonical loadings. Note that, depending on the variable, a negative loading does not necessarily reflect worse performance. ILF = inferior longitudinal fasciculus; SLFp = superior longitudinal fasciculus (parietal bundles); SLFt = superior longitudinal fasciculus (temporal bundles); UNC = uncinate fasciculus; MLU = mean length of utterance (in words); SI = subordination index; Clauses/Ut = mean number of clauses per utterance; WPM = words per minute; MATTR = moving-average type-token ratio; % Maze words = percentage of maze words; Wd dys/Ut = word-level dysfluencies per utterance; % Main events = proportion of main events; CIUs/min = correct information units per minute.

Multivariate outlier analysis using Mahalanobis distance identified four participants as lying significantly outside the sample distribution on the included spoken language and imaging variables (*P*  *<* 0.001).^64^ These four individuals are characterized in Supplementary Table 3. Three of these participants had abnormally high MD and/or low FA in at least one tract, and (separately) three of these participants had abnormally high word-level dysfluencies (among other deviations from the cohort as a whole). To examine the impact of the outlying participants, the CCAs were repeated with the outliers removed. The first set of canonical variates for FA was statistically significant (*n* = 127, *r_c_* = 0.51, *P*  *=* 0.041), as was the case for MD (*n* = 127, *r_c_* = 0.56, *P*  *=* 0.011). In contrast, none of the canonical variates for WMH volume were statistically significant (*n* = 127, *r_c_* = 0.41, *P*  *=* 0.80). Additionally, using a total anomaly volume that included WMH, strokes, lacunes, and perivascular spaces did not change the results (CCA was not statistically significant: *n* = 127, *r*_c_ = 0.47, *P* = 0.26). Comparing the canonical loadings of each white matter tract and spoken language variable for the first set of canonical variates for FA and MD before (Supplementary Fig. 2) and after (Fig. 3) outlier removal, it appeared the outliers had a noticeable influence on several of the variables’ canonical loadings (particularly FA and MD of right hemisphere tracts). Therefore, the remainder of the analyses excluded the four outlying participants.

Heatmaps of the cross-correlation matrices generated as part of the CCAs of WMH volume, FA, and MD are shown in Supplementary Fig. 3. The correlation strengths, eigenvalues, and statistical significance of each root (i.e. set of canonical variates) of these CCAs are provided in Supplementary Fig. 4. Only the first roots were significant in both the FA and MD CCAs. Therefore, only these first roots were used to explain the association between individual white matter tracts and spoken language performance.

The canonical loadings of each variable were used to interpret the relationship between white matter tract integrity and spoken language performance for the canonical variates that were statistically significant (Fig. 3). Considering the CCA for FA (Fig. 3A), the left SLF parietal and temporal bundles had the strongest loadings onto the white matter variate. These loadings were positive, representing an increase in FA values. FA in the left ILF, left UNC and right UNC also had moderate loadings—though, interestingly, the loadings for the UNC were negative. Within the spoken language variate, variables with considerable loadings included words per minute (productivity), percentage of maze words (% Maze words; fluency), word-level dysfluencies per utterance (Wd dys/Ut; fluency), the proportion of main events (% Main events; information content), and CIUs per min (CIUs/min; information content)—these five variables had loading directions consistent with improved performance. However, the subordination index (SI; syntax) and mean number of clauses per utterance (Clauses/Ut; syntax) had moderate negative loadings, suggesting surprisingly that higher FA in the left ILF, SLFp, and SLFt was associated with lower syntactic complexity. Mean length of utterance (MLU; syntax), moving-average type-token ratio (MATTR; lexical diversity), and total words (productivity) were not associated with FA in the eight white matter tracts we investigated.

The CCA for MD (Fig. 3B) similarly had the left SLF parietal and temporal bundles as the strongest loading white matter tracts in the white matter variate. Loadings of other tracts were weak but included small contributions from the left UNC and right SLF parietal and temporal bundles. The loading directions of both SLF bundles were negative, representing decreased MD, while the loading direction of the left UNC was positive. The spoken language variate included moderate loadings from Wd dys/Ut (fluency), % Main events (information content), and MLU (syntax), as well as a weaker loading from MATTR (lexical diversity). All these spoken language measures had loading directions consistent with improved performance. SI (syntax), Clauses/Ut (syntax), total words (productivity), words per minute (productivity), % Maze words (fluency), and CIUs/min (information content) were not substantially associated with MD in the eight white matter tracts investigated in this study.

Overall, white matter tracts in the left hemisphere loaded more strongly than those in the right hemisphere, with the left SLF parietal and temporal bundles having the strongest loadings of the eight tracts considered. Additionally, of the 10 spoken language measures, those related to syntax, fluency, and information content loaded most strongly.

### Sex and DTI-spoken language association

**Figure 4:**
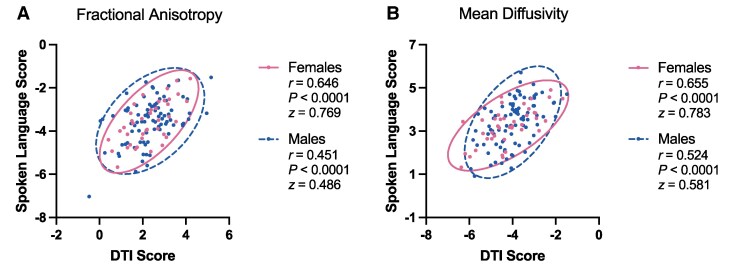
**Canonical variate scores of females and males.** Canonical variate scores from the CCAs of diffusion tensor imaging (DTI; Variate 1) and spoken language performance (Variate 2) were calculated for each participant (represented as individual dots). The scores of females (*n* = 41) and males (*n* = 86) were then correlated separately to examine potential sex differences in the association between DTI and spoken language. These sex-specific variate score correlations (both Pearson *r* and Fisher *z*-scores), along with 95% confidence ellipses, are shown for the CCAs of (**A**) fractional anisotropy and (**B**) mean diffusivity. Males and females had similarly strong correlations for both FA (*z* = 1.442, *P* = 0.149) and MD (*z* = 1.032, *P* = 0.302).

Correlations between DTI variate scores and spoken language variate scores, along with 95% confidence ellipses for each sex, for both fractional anisotropy and mean diffusivity are shown in Fig. 4. Considering the CCA of FA (Fig. 4A), females and males had similarly strong associations between DTI and spoken language scores (females: *n* = 41, *r* = 0.646, *P* < 0.0001, *z* = 0.769; males: *n* = 86, *r* = 0.451, *P* < 0.0001, *z* = 0.486; difference: *z* = 1.442, *P* = 0.149). Considering the CCA of MD (Fig. 4B), females and males again had similarly strong associations between DTI and spoken language scores (females: *n* = 41, *r* = 0.655, *P* < 0.0001, *z* = 0.783; males: *n* = 86, *r* = 0.524, *P* < 0.0001, *z* = 0.581; difference: *z* = 1.032, *P* = 0.302).

## Discussion

In this cohort study of people with cerebrovascular disease and varying levels of cognitive impairment, CCAs showed that both FA and MD in the NAWM of the left and right SLFp, SLFt, ILF, and UNC were significantly correlated with spoken language measures, while normalized WMH volume was not. These associations were found in participants following stroke (many with covert or silent strokes) whose post-stroke neurological exams were unremarkable for significant aphasia, dysarthria, or apraxia. Most of the participants (89%) were right-handed. The parietal and temporal bundles of the left SLF had the strongest canonical loadings of the eight white matter tracts investigated, and spoken language measures related to syntax, fluency, and information content frequently had the strongest canonical loadings of the 10 spoken language variables investigated. Female and male participants exhibited similarly strong associations of FA and MD with spoken language.

### Associations of white matter with spoken language

Higher values of FA and lower values of MD are generally associated with the preservation of white matter microstructure.^23^ Higher FA, particularly in the left SLFp and SLFt, corresponded to better performance on measures of fluency (lower percentage maze words, fewer word-level dysfluencies per utterance) and information content (higher proportion of main events, higher CIUs per minute) but less syntactic complexity (lower SI, fewer clauses per utterance). In addition, lower MD, particularly in the left SLFp and SLFt, corresponded to better performance on measures of fluency (fewer word-level dysfluencies per utterance), information content (higher proportion of main events), and more syntactic complexity (higher MLU). In contrast, the current study did not find that normalized tract lesion (WMH) volume was associated with spoken language performance. This adds to previous literature supporting the idea that the microstructure of NAWM may be more reflective of cognitive function than assessments of lesioned white matter,^30,65^ at least in people with cerebrovascular disease without significant aphasia or dysarthria. However, it is important to note that WMH volumes in the CVD cohort were quite low due to the inclusion/exclusion criteria, which aimed to select participants at risk for vascular dementia due to lacunar infarcts, small (non-disabling) strokes, and covert strokes. The limited range of severity of both WMH and spoken language impairments in this cohort could make it difficult to establish associations between these two factors.

The current study, with its examination of the association between DTI metrics in NAWM and spoken language, significantly expands our understanding of the neural underpinnings of language changes in individuals with covert or subclinical stroke without significant aphasia or dysarthria. This work is critical for elucidating cognitive impairment and risks for developing vascular cognitive impairment in people with CVD. Previous studies have used lesion-based approaches or DTI to examine neural correlates of spoken language at the level of discourse/connected speech in other cohorts. These studies include participants with post-stroke aphasia,^66–70^ primary progressive aphasia,^8,11,24,27,28^ Alzheimer’s disease spectrum,^7^ and healthy adults.^25,71^ While the findings of these studies are inevitably quite varied due to differences in underlying pathologies, they support the key role of the superior longitudinal/arcuate fasciculus in spoken language—particularly for productivity (e.g. words per minute,^66,67^ number of content words,^66^ number of words^24,68^), syntax (MLU,^69^ proportion of grammatically well-formed utterances^28^), information content (%CIUs,^67^ CIUs/min^67^), fluency (semantically related and unrelated paraphasias,^70^ number and time of pauses,^24^ number of words including mazes^24^), and lexical diversity (e.g. type-token ratio,^8^ words used once^8^). They also support the role of the ILF in syntax (proportion of grammatically well-formed utterances^28^), fluency (semantically related and unrelated paraphasias^70^), and lexical diversity (e.g. type-token ratio,^8^ words used once^8^). Finally, they demonstrate the role of the UNC in information content (narrative production^7^), lexical sophistication (e.g. age of acquisition, familiarity^8^), and fluency (e.g. number and time of pauses, number of words including mazes^24^). However, some studies did not find significant correlations between spoken language measures and DTI metrics in the ILF,^11,25^ SLF/arcuate^11,25^ or UNC.^11,27,67^

The current study is distinguished by its comprehensive approach to measuring the neural underpinnings of spoken language performance. Historically, the spoken language variables included in DTI studies have been limited, with few studies including spoken language measures of syntax complexity, word and sound-level errors, and event-level measures of information content. Unexpectedly, we found that positive loadings of FA in the DTI variate corresponded to negative loadings on spoken language measures of syntax complexity (SI and mean clauses per utterance). In contrast, previous studies in primary progressive aphasia have found positive correlations between FA in the left SLF and syntactic comprehension^72^ and production.^24,28,69,72^ In general, neurolinguistic research suggests that syntactic processes may be mediated by both dorsal (SLF/arcuate) and ventral pathways (UNC, extreme capsule),^73,74^ but that dorsal pathways are necessary for processing sentences with higher syntactic complexity.^75^ Interestingly, a study of healthy participants by Antenenko *et al*.^71^ found that syntactic comprehension correlated with FA in the left SLF in young adults but not older adults. Instead, syntactic comprehension correlated with FA in the left UNC in older adults but not young adults, suggesting a reorganization of syntax networks even in healthy aging that could intersect with disease-related changes.

One possible interpretation of the current study’s finding that requires further examination in future studies is that in spontaneous language in persons with CVD, preserved white matter microstructure in the left SLF parietal and temporal bundles is associated with a strategic and possibly compensatory reduction in syntax complexity. We hypothesize that the formation of simpler syntax structures in CVD may help reduce the overall cognitive load on the spoken language system, allowing these individuals to preserve function for other aspects of their spoken language production, such as fluency and information content. Similar hypotheses have been posited for reduced syntax complexity in spontaneous language in Huntington’s disease^76^ and aphasia secondary to stroke.^77,78^ If accurate, this would suggest that a more intact white matter microstructure supports the overall optimization of spoken language production.

However, it is also important to consider that increased FA is not always beneficial; for example, visuospatial deficits in William’s syndrome have been associated with increased FA in the right SLF.^79^ Several factors could lead to increased FA, including increased myelination and axonal density or decreased axonal diameter, membrane permeability, and branching.^80–82^ Therefore, the negative loading of syntax performance could be due to abnormal increases in FA in the SLFp, SLFt, or ILF. In line with this idea, the Pearson correlations of the left SLFt, left SLFp, and ILF with mean clauses per utterance were negative, although not statistically significant. A caveat of this theory is that if the increased FA in the SLFp, SLFt, and ILF was maladaptive, then worse performance in the other domains of spoken language would also have been expected. Additionally, previous literature in people with CVD aetiologies, including chronic stroke^83,84^ and the ONDRI CVD cohort,^51^ report lower FA and higher MD relative to healthy controls^83^ or non-lesioned brain regions.^51,83,84^

In contrast to the unexpected FA-syntax relationship, decreased MD in the DTI variate corresponded to higher syntactic complexity (with respect to MLU). Overall, there were two notable differences between the CCAs of FA and MD. First, the CCA of MD had a slightly higher canonical correlation strength than that of FA (*r_c_* = 0.557 versus 0.509). Second, while both the FA and MD CCAs had the strongest spoken language variate loadings from syntax, fluency and information content measures, FA also had a considerable loading from words per minute, and MD additionally had a considerable loading from MATTR.

Within white matter, MD is affected by cellular and membrane density and can increase due to disease processes like oedema or necrosis.^85^ The voxel resolution in DTI (2 mm isotropic in the current study) is large compared to the diameter of an axon. Consequently, a single voxel can contain a variety of fibre populations that are oriented in different directions.^82^ Crossing fibres within a voxel can confound the effects of microstructural changes due to white matter degeneration and lead to the underestimation of FA values. However, MD is less affected by fibre crossing than FA.^86^ This may explain why MD was slightly more sensitive to spoken language performance than FA.

Some variables did not load strongly in the current study. The right ILF consistently had negligible loadings and was therefore not found to be associated with spoken language performance in our CCAs. This may be related to the fact that the cohort was predominantly right-handed, or this could be due to the choice of spoken language variables and domains included in this study rather than a lack of importance of the right ILF for spoken language in general. The right ILF has previously been shown to relate to measures of lexical sophistication, such as age of acquisition and noun frequency,^8^ but the present study did not include any measures of lexical sophistication.

Other studies on the neural correlates of spoken language production have highlighted significant associations with white matter regions such as the inferior fronto-occipital fasciculus,^7,26,28,70^ frontal aslant tract,^27,66^ posterior thalamic radiation,^70^ retrolenticular part of the internal capsule,^70^ and posterior corona radiata,^70^ which were not included in the version of TRACULA^35^ used in the current study. Future work should examine the role of these additional white matter pathways in spoken language and cerebrovascular disease. That said, the narrow focus of the current study was deliberate. Adding additional variables to CCAs can reduce their interpretability. CCAs forgo the simpler interpretation of bivariate associations. However, CCA is advantageous because it considers the relationships between all the variables simultaneously and exploits more variability in the data than bivariate correlations. Therefore, CCA can detect associations that might be hidden from traditional bivariate correlations. This effect was demonstrated by our observation of substantial loadings of our variables in the CCAs despite observing no significant Pearson correlations between the DTI and spoken language variables after false discovery rate correction (Supplementary Fig. 3). This extra statistical power was important, given the cohort was intentionally sampled to minimize severe pathology and clinical symptoms.

### Sex and DTI-spoken language associations

Neither FA nor MD in the eight white matter tracts studied differed significantly between female and male participants, consistent with previous studies examining sex differences in DTI measures following stroke or cerebrovascular disease.^25,87^ We observed that females had significantly higher WMH volumes than males in the left UNC, right SLFt, and right SLFp. While studies by Etherton *et al.*^87^ and Bonkhoff *et al.*^88^ did not find significant sex differences in whole-brain WMH volume following acute ischaemic stroke, they did not investigate individual tracts.

In the current study, female and male participants had similarly strong associations between spoken language and NAWM microstructure. However, a limitation inherent in this sex-based comparison is the relatively small cohort of female participants (41 compared to 86 male participants). There exists a long-debated theory that women have more bilateral language representation than men^89–93^ or that sex mediates the effect of aging on the lateralization of language.^94^ However, the current study (which included tracts in both hemispheres) does not support this. Overall, more sex-based analyses of neurolinguistics using DTI will be needed to resolve this debate.

### Strengths and Limitations

To our knowledge, this is the first study examining the association between spoken language parameters and the integrity of the NAWM portion of white matter tracts involved in language pathways in people at elevated risk for dementia due to cerebrovascular disease. We also evaluated the potential association between the lesioned portion of these tracts with spoken language performance. This is crucial because we believe that NAWM plays a more substantial role in cognitive processes than lesioned white matter. Therefore, NAWM may more precisely reflect language performance. Additionally, this study included a well-characterized cohort of cerebrovascular disease patients much larger than most previous studies of neural correlates of spoken language.

However, several limitations to this study must be considered. First, a healthy control group was not included. Such a control group could have focused the CCA on tracts that differed in the CVD cohort. Second, the handedness of our participants was not controlled but would likely play a role in the lateralization of language. Given that our cohort was predominantly (89%) right-handed, it is unsurprising that left hemisphere tracts loaded most strongly in this study. Additionally, DTI is very sensitive to changes in white matter microstructure, but it is not specific to a particular mechanism of microstructural change. Novel diffusion MRI metrics such as microscopic fractional anisotropy may provide a more sensitive avenue for white matter microstructure investigations in the future.^95^ Finally, as mentioned, the SLFp and SLFt shared 8.0% and 4.5% of voxels in the left hemisphere, which may have contributed in part to their similar canonical loadings. This study also included limited white matter tracts compared to what previous studies have demonstrated are associated with spoken language production,^7,26–28,66,70^ though this was necessary to maintain statistical power for the CCA approach.

## Conclusion

This study examined white matter hyperintensity volume and NAWM microstructural correlates of spoken language in a relatively large, robustly characterized cohort of individuals with cerebrovascular disease at elevated risk for vascular cognitive impairment and dementia. Most participants had subclinical or very mild strokes. Symptoms of aphasia and motor speech disorders were absent or very mild at the time of stroke presentation. DTI measures indicated that altered microstructure in the NAWM of primarily the left superior longitudinal fasciculus was associated with altered spoken language performance, whereas tract hyperintensity volume was not. These results demonstrate the importance of DTI and tractography for evaluating the neurological mechanisms of spoken language impairment and reinforce the idea that there may be mild or early-stage damage in NAWM that can be detected through DTI but not traditional structural MRI. Based on this demonstrated sensitivity, DTI in NAWM may be particularly important for evaluating neurological mechanisms in people with relatively mild cerebrovascular pathology (as opposed to those with severe stroke). The current study also showed that multi-domain spoken language analysis is sensitive to underlying white matter microstructure even in individuals without significant aphasia, supporting its value as a tool for assessing cognitive impairment in neurodegenerative disease. Whether spoken language changes precede detectable changes in white matter requires further investigation. Given the widespread burden of cerebrovascular pathology within all dementia aetiologies, we anticipate this work will inform future investigations into the mechanisms of spoken language and cognitive impairment in various neurodegenerative diseases.

## Supplementary Material

fcaf145_Supplementary_Data

## Data Availability

ONDRI study data are available from the Ontario Brain Institute upon submission of a study proposal and approval for access (https://www.braincode.ca/). The specific datasets used in this study are listed in the Appendix. The custom MatLab code used to exclude lesions from the white matter tracts is provided in the Supplementary Materials.

## Appendix

### ONDRI Investigators

Agessandro Abrahao, Sabrina Adamo, Derek Beaton, Sandra Black, Alanna Black, Michael Borrie, Don Brien, Susan Bronskill, Dennis Bulman, Brian Coe, Ben Cornish, Sherif Defrawy, Jane Lawrence Dewar, Allison Ann Dilliott, Roger A. Dixon, Sali Farhan, Frederico Faria, Elizabeth Finger, Corinne Fischer, Andrew Frank, Julia Fraser, Morris Freedman, Mahdi Ghani, Barry Greenberg, David Grimes, Wendy Hatch, Rob Hegele, Melissa Holmes, Chris Hudson, Mandar Jog, Peter Kleinstiver, Sanjeev Kumar, Donna Kwan, Tony Lang, Elena Leontieva, Brian Levine, Wendy Lou, Efrem Mandelcorn, Jennifer Mandzia, Ed Margolin, Connie Marras, Mario Masellis, Bill McIlroy, Manuel Montero-Odasso, Doug Munoz, David Munoz, Miracle Ozzoude, Stephen Pasternak, Bruce Pollock, Tarek Rajji, Natalie Rashkovan, John Robinson, Ekaterina Rogaeva, Demetrios Sahlas, Yanina Sarquis Adamson, Dallas Seitz, Christen Shoesmith, Alisia Southwell, Tom Steeves, Michael Strong, Stephen Strother, Sujeevini Sujanthan, Kelly Sunderland, Brian Tan, David Tang-Wai, Carmela Tartaglia, Faryan Tayyari, Athena Theyers, John Turnbull, Karen Van Ooteghem, John Woulfe, Modjeh Zamyadi and Lorne Zinman.

### ONDRI datasets used in this study

*Clinical Release:*

Demographics—OND01_VCI_01_CLIN_DEMOG

Disease-specific history—OND01_VCI_01_CLIN_DHX

Medical history checklist—OND01_VCI_01_CLIN_MEDHX

Montreal Cognitive Assessment—OND01_VCI_01_CLIN_MOCA

Modified Rankin Scale—OND01_VCI_01_CLIN_MRS

NIH Stroke Scale—OND01_VCI_01_CLIN_NIHSS

Smoking history—OND01_VCI_01_CLIN_SMOKE

*Neuropsychology release:*

Sequenced Story Task—OND01_VCI_01_NPSY_SEQSTORT2A (note that information content variables, including CIUs and % main events, are not included but may be made available on request to Dr. Angela C. Roberts: angela.roberts@uwo.ca)

*Neuroimaging release:*

T_1_-weighted—OND01_VCI_01_NIMG_DEFACEDT1

Diffusion—OND01_VCI_01_NIMG_DTI

SABRE-LE—3D SABRE-LE images are currently not available. However, tabular ROI data of the extracted brain regions is available in OND01_VCI_01_NIMG_SABR_FULL, and the FLAIR and PD/T_2_-weighted images used to generate the SABRE-LE images are available in OND01_VCI_01_NIMG_DEFACEDFLAIR and OND01_VCI_01_NIMG_DEFACEDPDT2.
