# Supplementary material for: White matter tract correlations with spoken language in cerebrovascular disease
Source: Brain Commun. 2025 Apr 19;7(3):fcaf145. doi: 10.1093/braincomms/fcaf145 (PMC12062522; doi:10.1093/braincomms/fcaf145)
Supplement: fcaf145_Supplementary_Data [file fcaf145_supplementary_data.pdf]

Supplementary Materials

Supplementary Tables & Figures

Supplementary Table 1. Additional clinical characteristics.

	**Females** (*n* = 44)		**Males** (*n* = 87)	
Continuous variable<br>OR<br>Categorical/ordinal variable	Median (IQR)<br><br>*n*	Range<br><br>%	Median (IQR)<br><br>*n*	Range<br><br>%
**Stroke history**				
TOAST classification of presumed stroke etiology				
Small artery occlusion (lacune)	16	36.4%	25	28.7%
Large artery atherosclerosis	7	15.9%	22	25.3%
Cardioembolic	6	13.6%	14	16.1%
Undetermined etiology	12	27.3%	19	21.8%
Other determined etiology	3	6.8%	3	3.4%
**Medical history (n, % yes)**				
Prior history of stroke	8	18.2%	14	16.1%
Prior history of transient ischemic attack	4	9.1%	16	18.4%
Prior history of intracranial hemorrhage	0	0.0%	1	1.1%
Hypertension	32	72.7%	62	71.3%
Coronary artery disease	4	9.1%	19	21.8%
Diabetes	7	15.9%	23	26.4%
Prior history of smoking	19	43.2%	55	63.2%
**NIHSS****				
Language score				
% No aphasia	41	100.0%	81	97.6%
% Mild to moderate aphasia	0	0.0%	2	2.4%
Dysarthria score				
% No dysarthria	39	95.1%	82	98.8%
% Mild to moderate dysarthria	2	4.9%	1	1.2%
Total score	0.0 (1.0)	0-6	0.0 (1.0)	0-3

IQR = interquartile range; TOAST = Trial of Org 10172 in Acute Stroke Treatment[1]; NIHSS = National Institutes of Health Stroke Scale.[2] **NIHSS scores were not available for seven participants who were transferred from the AD/MCI cohort to the CVD cohort. Therefore, percentages are calculated out of 124 participants (female: *n* = 41, male: *n* = 83).

Supplementary Table 2. Infarct and lacune prevalence by region. Prevalence was determined by counting all subjects with non-zero lacune or infarct volumes within each region in the SABRE-LE images.

		Prevalence	
**Anomaly**	**Region**	**# of participants**	**% of participants**
**Lacunes**	Left Superior Frontal (Lateral)	14	10.7
	Left Superior Frontal (Medial)	7	5.3
	Left Middle Frontal (Lateral)	67	51.1
	Left Middle Frontal (Medial)	52	39.7
	Left Inferior Frontal (Lateral)	19	14.5
	Left Inferior Frontal (Medial)	19	14.5
	Left Superior Parietal	11	8.4
	Left Inferior Parietal	68	51.9
	Left Occipital	32	24.4
	Left Anterior Temporal	9	6.9
	Left Posterior Temporal	45	34.4
	Left Anterior Basal Ganglia and Thalamus	47	35.9
	Left Posterior Basal Ganglia and Thalamus	40	30.5
	Left Hippocampus	0	0.0
	Right Superior Frontal (Lateral)	7	5.3
	Right Superior Frontal (Medial)	9	6.9
	Right Middle Frontal (Lateral)	78	59.5
	Right Middle Frontal (Medial)	69	52.7
	Right Inferior Frontal (Lateral)	15	11.5
	Right Inferior Frontal (Medial)	33	25.2
	Right Superior Parietal	11	8.4
	Right Inferior Parietal	76	58.0
	Right Occipital	34	26.0
	Right Anterior Temporal	5	3.8
	Right Posterior Temporal	42	32.1
	Right Anterior Basal Ganglia and Thalamus	49	37.4
	Right Posterior Basal Ganglia and Thalamus	33	25.2
	Right Hippocampus	0	0.0
	Any Region	121	92.4
**Infarcts**	Left Superior Frontal (Lateral)	23	17.6
	Left Superior Frontal (Medial)	7	5.3
	Left Middle Frontal (Lateral)	16	12.2
	Left Middle Frontal (Medial)	3	2.3
	Left Inferior Frontal (Lateral)	3	2.3
	Left Inferior Frontal (Medial)	0	0.0
	Left Superior Parietal	22	16.8
	Left Inferior Parietal	32	24.4
	Left Occipital	23	17.6
	Left Anterior Temporal	3	2.3
	Left Posterior Temporal	22	16.8
	Left Anterior Basal Ganglia and Thalamus	0	0.0
	Left Posterior Basal Ganglia and Thalamus	1	0.8
	Left Hippocampus	0	0.0
	Right Superior Frontal (Lateral)	13	9.9
	Right Superior Frontal (Medial)	6	4.6
	Right Middle Frontal (Lateral)	14	10.7
	Right Middle Frontal (Medial)	7	5.3
	Right Inferior Frontal (Lateral)	6	4.6
	Right Inferior Frontal (Medial)	1	0.8
	Right Superior Parietal	16	12.2
	Right Inferior Parietal	23	17.6
	Right Occipital	19	14.5
	Right Anterior Temporal	7	5.3
	Right Posterior Temporal	22	16.8
	Right Anterior Basal Ganglia and Thalamus	6	4.6
	Right Posterior Basal Ganglia and Thalamus	4	3.1
	Right Hippocampus	0	0.0
	Any Region	73	55.7

Supplementary Table 3. Characteristics of participants identified as statistically significant multivariate outliers. Four participants were identified as statistically significant multivariate outliers ($P < 0.001$) using the Mahalanobis distance evaluated with a chi-squared distribution ($df = 34$). Age ranges are provided instead of a specific age to maintain anonymity. Clauses / Ut = mean number of clauses per utterance; FA = fractional anisotropy; ILF = inferior longitudinal fasciculus; MD = mean diffusivity; MLU = mean length of utterance (in words); mRS = modified Ranking Scale[3]; MoCA = Montreal Cognitive Assessment[4]; NIHSS = National Institutes of Health Stroke Scale[2]; NAWM = normal-appearing white matter; SI = subordination index; SLFp = superior longitudinal fasciculus (parietal bundles); SLFt = superior longitudinal fasciculus (temporal bundles); UNC = uncinate fasciculus; WMH = white matter hyperintensities; Wd dys / Ut = word-level dysfluencies per utterance.

**Outlying Participant**	**1**	**2**	**3**	**4**
Sex	Male	Female	Female	Female
Approximate age	65-75	65-75	75-85	55-65
Education (years)	16	16	20	16
Handedness	Right	Right	Right	Right
mRS total score	0	2	1	0
MoCA total score	28	28	23	22
NIHSS language score	No aphasia	No aphasia	No aphasia	No aphasia
NIHSS dysarthria score	No dysarthria	No dysarthria	No dysarthria	No dysarthria
NIHSS total score	1	0	0	0
NAWM (cm^3)	341.1	330.6	325.6	344.0
WMH (cm^3)	47.1	55.2	1.2	1.5
Lacunar volume (mm^3)	3454	2066	18	120
Stroke lesion volume (cm^3)	0.0	0.0	2.1	25.3
p-value of Mahalanobis distance	0.0001	0.0003	0.0004	0.0008
**Variables with outlying observations**[a]				
Extremely high	MD left ILF<br>MD left SLFp<br>MD left UNC<br>MD right ILF<br>MD right SLFt<br>MD right UNC	MD left SLFp	MLU<br>Clauses / Ut<br>Wd dys / Ut	Wd Dys / Ut
High	Wd dys / Ut<br>MD right SLFp	MD left SLFt<br>MD left UNC<br>MD right SLFp<br>MD right SLFt<br>MD right UNC	SI	MD right SLFt
Low	FA left SLFp<br>FA left SLFt<br>FA right ILF<br>FA right UNC	FA left SLFp	n/a	n/a
Extremely low	FA left ILF	n/a	n/a	n/a

[a]Outlying observations were classified as follows:

Extremely high > 3rd Quartile + 3 × IQR

High > 3rd Quartile + 1.5 × IQR

Low < 1st Quartile – 1.5 × IQR

Extremely low < 1st Quartile – 3 × IQR

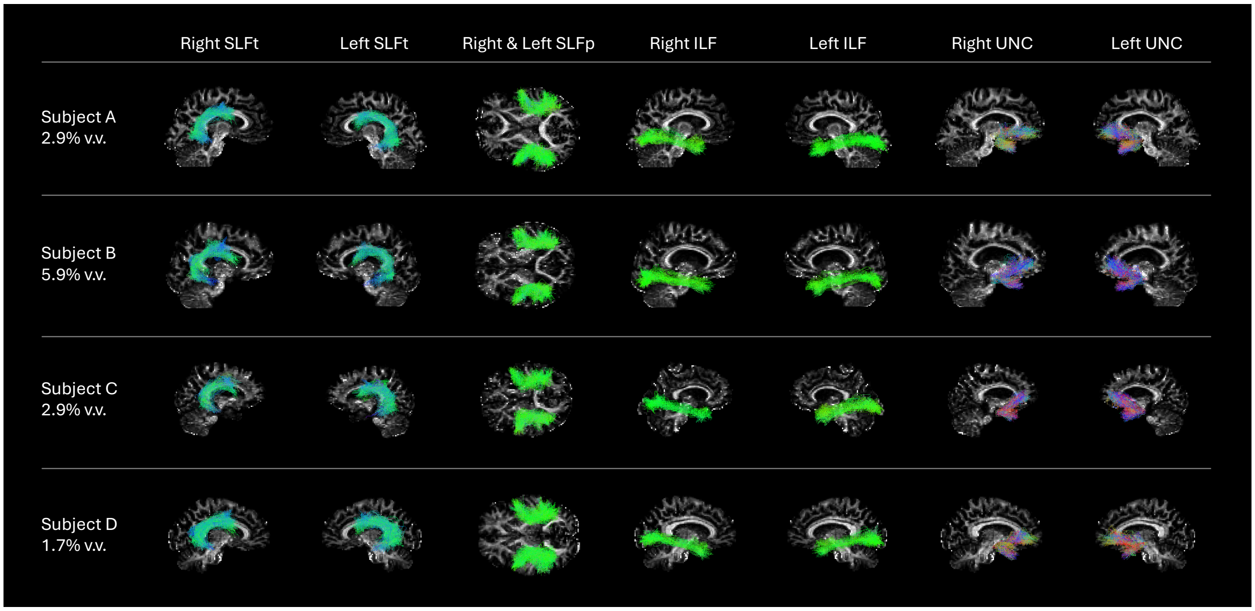

Supplementary Figure 1. Visualization of pathway streamlines. Global probabilistic tractography with anatomical neighbourhood priors was performed by TRACULA. The streamlines that were accepted by the tractography algorithm and then summed to estimate the posterior probability distribution for each white matter pathway are shown for four representative subjects from different imaging sites in the current study (colour indicates the orientation of each streamline's endpoints, RAS → RGB). Subjects from four different recruitment sites with diverse ventricular volumes (v.v., expressed as a percent of total intracranial volume) were deliberately chosen for this figure to show the ability of TRACULA to accommodate varying subject anatomy. Note that the track files output by TRACULA and used to visualize these streamlines are not smooth like typical streamlines in deterministic tractography; they contain only integer voxel coordinates and therefore have a stepladder appearance. Additionally, not conveyed here, posterior probability distributions for each pathway were thresholded at 50% of the maximum probability and lesion masks were used to remove all non-normal-appearing white matter voxels from the final white matter pathway segmentations (see Figure 2 in the main text).

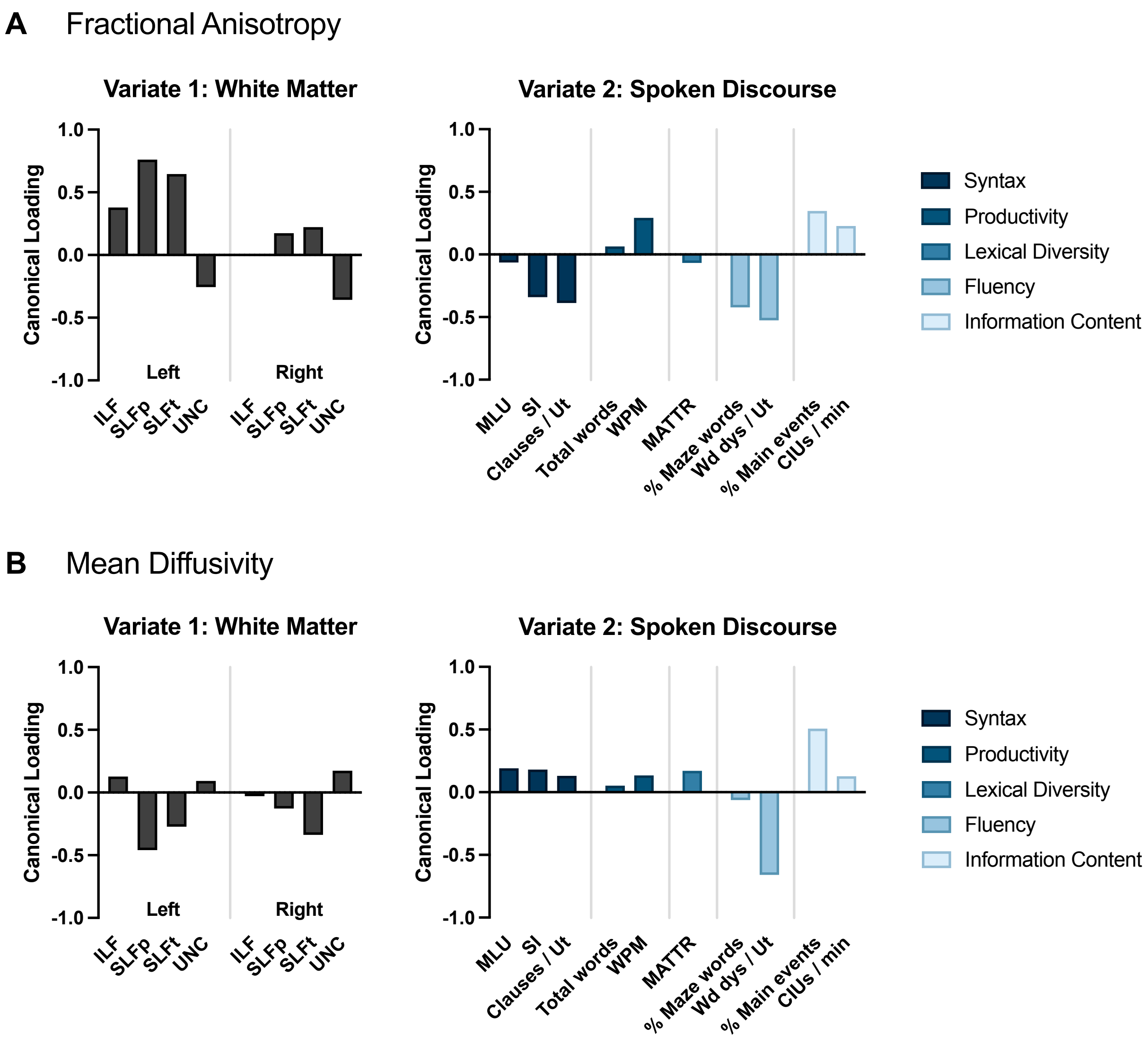

Supplementary Figure 2. Canonical correlation loadings of diffusion tensor imaging and spoken language measures in individuals with cerebrovascular disease prior to outlier removal. Canonical correlations examined the association between diffusion tensor imaging (DTI) metrics in both hemispheres of the brain and spoken language performance. The canonical loadings of each DTI and spoken language variable onto their respective variates are shown for (**A**) fractional anisotropy (n = 131, r_c = 0.510, P = 0.23) and (**B**) mean diffusivity (n = 131, r_c = 0.547, P = 0.016). As shown in the legends, spoken language variables can be categorized into several performance domains. Note that, depending on the variable, a negative loading does not necessarily reflect worse performance.

ILF = inferior longitudinal fasciculus; SLFp = superior longitudinal fasciculus (parietal bundles); SLFt = superior longitudinal fasciculus (temporal bundles); UNC = uncinate fasciculus; MLU = mean length of utterance (in words); SI = subordination index; Clauses / Ut = mean number of clauses per utterance; WPM = words per minute; MATTR = moving-average type-token ratio; % Maze words = percentage of maze words; Wd dys / Ut = word-level dysfluencies per utterance; % Main events = proportion of main events; CIUs / min = correct information units per minute.

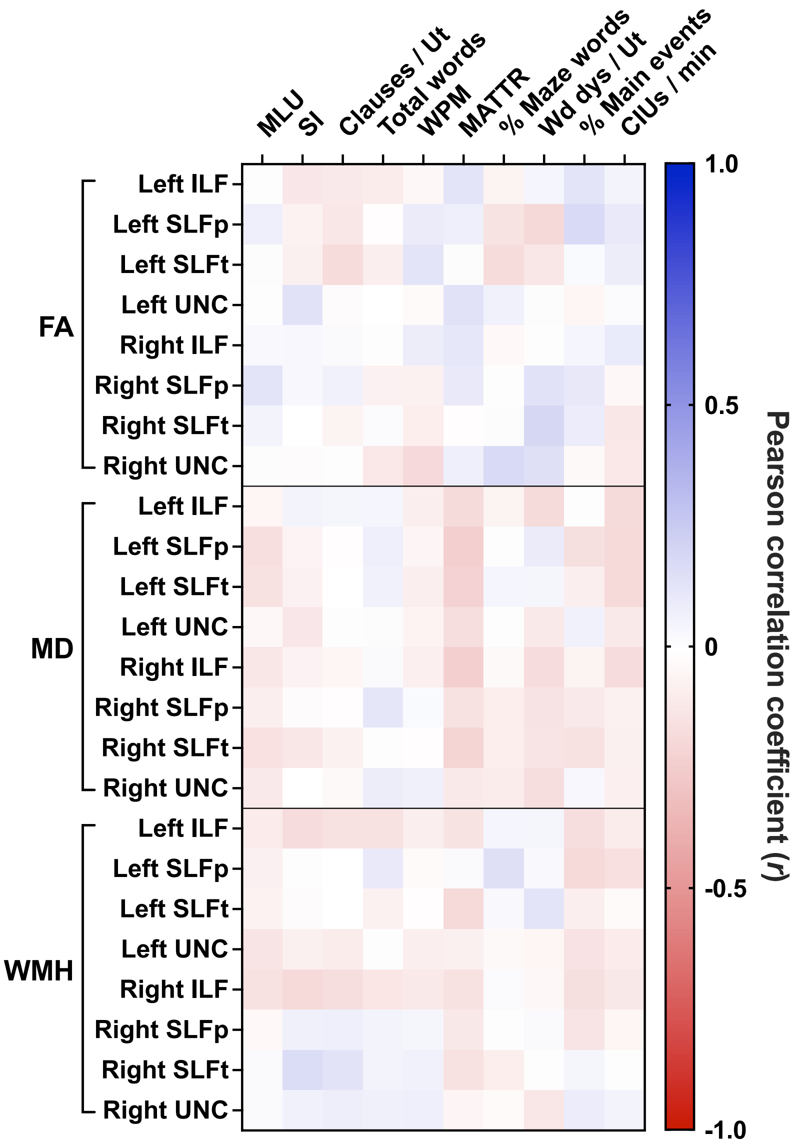

Supplementary Figure 3. Cross-correlation heatmaps of bivariate correlations between white matter and spoken language variables (*n* = 127). Pearson correlation coefficient (*r*) heatmaps are shown for the cross-correlations of spoken language variables with fractional anisotropy (FA), mean diffusivity (MD), and normalized white matter hyperintensity (WMH) volume in each white matter tract. None of these correlations were statistically significant after controlling for the false discovery rate (α = 0.05, q = 0.05), likely due to the relatively mild severity of white matter and spoken language alterations in this cohort.

ILF = inferior longitudinal fasciculus; SLFp = superior longitudinal fasciculus (parietal bundles); SLFt = superior longitudinal fasciculus (temporal bundles); UNC = uncinate fasciculus; MLU = mean length of utterance (in words); SI = subordination index; Clauses / Ut = mean number of clauses per utterance; WPM = words per minute; MATTR = moving-average type-token ratio; % Maze words = percentage of maze words; Wd dys / Ut = word-level dysfluencies per utterance; % Main events = proportion of main events; CIUs / min = correct information units per minute.

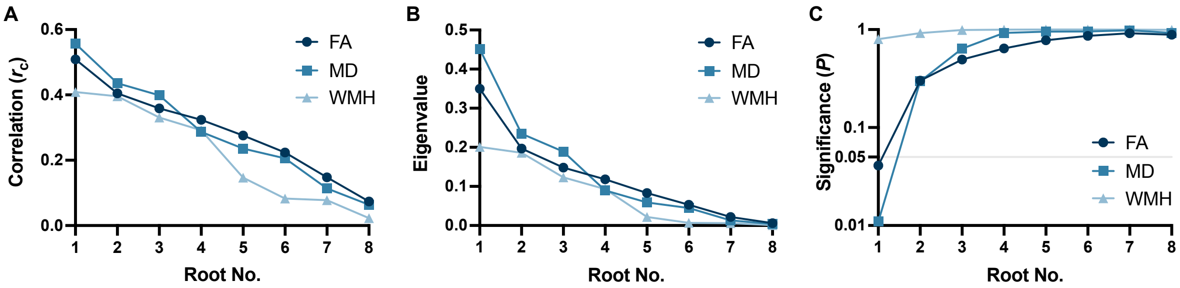

Supplementary Figure 4. Canonical correlation analyses characteristics. Three canonical correlation analyses (CCAs) examined the association between spoken language performance and white matter tract integrity in the form of fractional anisotropy (FA), mean diffusivity (MD), and white matter hyperintensity (WMH) volume (n = 127). (**A**) Canonical correlation strengths (r_c), (**B**) eigenvalues, and (**C**) statistical significance (p) are shown for each of the eight orthogonal canonical sets (roots) produced by the CCAs. Only the first roots of the FA and MD CCAs were statistically significant ($P < 0.05$).

Custom Code

MatLab script for excluding lesions and non-NAWM from FreeSurfer's TRACULA tract segmentations (as binary masks) using ONDRI's SABRE-LE images:

```
% *************MATLAB code for Extraction of the lesions and anomalies from WM tracts' segmentations generated by FreeSurfer (V. 6.0.0) TRACULA in a single subject and tract*************

% Note: This code is consistent with FreeSurfer and TRACULA Output V.6.0.0. For newer versions minor modifications in the computer codes and commands may be necessary considering the structure of the output folders of FreeSurfer/TRACULA.

% The code was written assuming that:

% 1) the FreeSurfer directory including FreeSurfer completed segmentation and TRACULA analysis in a single subject is "Subject_0" folder available in the MATLAB current directory, and

% 2) the ONDRI lesion mask in the single subject (as a NIFTI file) is "ONDRI_lesion_mask_Subject_0.nii.gz" available in the MATLAB current directory.

Note: Code can be modified and run for all other remaining 17 tracts (out of 18 tracts segmented by TRACULA) as completed for the example tract fmajor to calculate the mean DTI metrics in the non-lesioned part of those tracts.

%*********************************************************************************************

% #####Step 1: Converting the related T1-weighted MGZ files (typical imaging file format used by FreeSurfer software) to the corresponding T1-weighted NIFTI files usable by image processing software (such as FSL)

% Converting the original T1-weighted image with the original matrix size generated by MRI scanner

command_1 = 'mri_convert Subject_0/mri/orig/001.mgz Subject_0/mri/orig/001.nii.gz';

status_1 = system(command_1);  %Executing the system command from MATLAB

% Converting the T1-weighted image with the matrix size 256×256×256 generated by zero padding by FreeSurfer software
% Note: All the FreeSurfer segmentation and tractography analysis are completed using this version of T1-weighted imaging file in FreeSurfer software.

command_2 = 'mri_convert Subject_0/mri/orig/001.mgz Subject_0/mri/orig.nii.gz';

status_2 = system(command_2);

%*********************************************************************************************

% #####Step 2: Transferring the DTI maps from the DTI space to the native T1 space using the Boundary Based Registration (BBR) transformation generated by FreeSurfer software available in Subject_0/dmri/xfms/diff2anatorig.bbr.mat

% FA map

command_3 = 'flirt -in Subject_0/dmri/dtifit_FA.nii.gz -applyxfm -init Subject_0/dmri/xfms/diff2anatorig.bbr.mat -out Subject_0/dmri/dtifit_FA_toanatorig.nii.gz -paddingsize 0.0 -interp trilinear -ref Subject_0/mri/orig.nii.gz';

status_3 = system(command_3);

% L1 (AD) map

command_4 = 'flirt -in Subject_0/dmri/dtifit_L1.nii.gz -applyxfm -init Subject_0/dmri/xfms/diff2anatorig.bbr.mat -out Subject_0/dmri/dtifit_AD_toanatorig.nii.gz -paddingsize 0.0 -interp trilinear -ref Subject_0/mri/orig.nii.gz';

status_4 = system(command_4);

% L2 map

command_5 = 'flirt -in Subject_0/dmri/dtifit_L2.nii.gz -applyxfm -init Subject_0/dmri/xfms/diff2anatorig.bbr.mat -out Subject_0/dmri/dtifit_L2_toanatorig.nii.gz -paddingsize 0.0 -interp trilinear -ref Subject_0/mri/orig.nii.gz';
```

```
status_5 = system(command_5);

% L3 map

command_6 = 'flirt -in Subject_0/dmri/dtifit_L3.nii.gz -applyxfm -init
Subject_0/dmri/xfms/diff2anatorig.bbr.mat -out Subject_0/dmri/dtifit_L3_toanatorig.nii.gz -paddingsize
0.0 -interp trilinear -ref Subject_0/mri/orig.nii.gz';

status_6 = system(command_6);

% MD map

command_7 = 'flirt -in Subject_0/dmri/dtifit_MD.nii.gz -applyxfm -init
Subject_0/dmri/xfms/diff2anatorig.bbr.mat -out Subject_0/dmri/dtifit_MD_toanatorig.nii.gz -paddingsize
0.0 -interp trilinear -ref Subject_0/mri/orig.nii.gz';

status_7 = system(command_7);

%*******************************************************************************************

% #####Step 3: Generating RD map in the native T1 space from L2 (dtifit_L2_toanatorig.nii.gz) and L3
(dtifit_L2_toanatorig.nii.gz) maps already transferred to the native T1 space

command_8 = 'fslmaths Subject_0/dmri/dtifit_L2_toanatorig.nii.gz -add
Subject_0/dmri/dtifit_L3_toanatorig.nii.gz -mul 0.5 Subject_0/dmri/dtifit_RD_toanatorig.nii.gz';

status_8 = system(command_8);

%*******************************************************************************************

% #####Step 4: Resizing the matrix size of the DTI maps (FA, MD, AD, and RD) already transferred to the
native T1 space to make them consistent with the T1-weighted image original matrix size by removing the
zero padding applied by FreeSurfer software

% FA map

command_9 = 'mri_convert Subject_0/dmri/dtifit_FA_toanatorig.nii.gz
Subject_0/dmri/dtifit_FA_toanatorig_resized.nii.gz --like Subject_0/mri/orig/001.nii.gz';

status_9 = system(command_9);

% MD map

command_10 = 'mri_convert Subject_0/dmri/dtifit_MD_toanatorig.nii.gz
Subject_0/dmri/dtifit_MD_toanatorig_resized.nii.gz --like Subject_0/mri/orig/001.nii.gz';

status_10 = system(command_10);

% AD map

command_11 = 'mri_convert Subject_0/dmri/dtifit_AD_toanatorig.nii.gz
Subject_0/dmri/dtifit_AD_toanatorig_resized.nii.gz --like Subject_0/mri/orig/001.nii.gz';

status_11 = system(command_11);

% RD map

command_12 = 'mri_convert Subject_0/dmri/dtifit_RD_toanatorig.nii.gz
Subject_0/dmri/dtifit_RD_toanatorig_resized.nii.gz --like Subject_0/mri/orig/001.nii.gz';

status_12 = system(command_12);

%*******************************************************************************************

% #####Step 5: Transferring the WM tracts' segmentation masks (probabilistic spatial distributions)
generated by TRACULA from the DTI space to the native T1 space using the Boundary Based Registration
(BBR) transformation generated by FreeSurfer software available in
Subject_0/dmri/xfms/diff2anatorig.bbr.mat

% Transferring fmajor: Corpus callosum - forceps major

command_13 = 'flirt -in Subject_0/dpath/fmajor_PP_avg33_mni_bbr/path.pd.nii.gz -applyxfm -init
Subject_0/dmri/xfms/diff2anatorig.bbr.mat -out
```

```
Subject_0/dpath/fmajor_PP_avg33_mni_bbr/path.pd_toanatorig.nii.gz -paddingsize 0.0 -interp trilinear -ref Subject_0/mri/orig.nii.gz';

status_13 = system(command_13);

%*******************************************************************************************

% #####Step 6: Resizing the matrix size of the WM tracts' segmentation masks (probabilistic spatial distributions) already transferred to the native T1 space to make them consistent with the T1-weighted image original matrix size by removing the zero padding applied by FreeSurfer software

% Resizing fmajor: Corpus callosum - forceps major

command_14 = 'mri_convert Subject_0/dpath/fmajor_PP_avg33_mni_bbr/path.pd_toanatorig.nii.gz Subject_0/dpath/fmajor_PP_avg33_mni_bbr/path.pd_toanatorig_resized.nii.gz --like Subject_0/mri/orig/001.nii.gz';

status_14 = system(command_14);

%*******************************************************************************************

% #####Step 7: Generating the WM tracts' segmentation binary masks from the resized WM tracts' probabilistic spatial distribution masks

% fmajor: Corpus callosum - forceps major

% Reading the maximum value of the resized probabilistic spatial distribution mask of fmajor

fmajor_path_pd=niftiread('Subject_0/dpath/fmajor_PP_avg33_mni_bbr/path.pd_toanatorig_resized.nii.gz');

max_fmajor_path_pd=max(fmajor_path_pd(:));

% Considering half of the maximum value of the probabilistic spatial distribution mask of fmajor for thresoding (%50 thresholding)

threshold_fmajor_50=0.5*max_fmajor_path_pd;

% Generating the binary mask for the tract fmajor form the thresholded probabilistic spatial distribution mask of fmajor (%50 thresholding)

command_15 = 'fslmaths Subject_0/dpath/fmajor_PP_avg33_mni_bbr/path.pd_toanatorig_resized.nii.gz -thr threshold_fmajor_50 -bin Subject_0/dpath/fmajor_PP_avg33_mni_bbr/path.pd_toanatorig_resized_threshold_50_bin.nii.gz';

status_15 = system(command_15);

%*******************************************************************************************

% #####Step 8: Generating normal-appearing white matter (NAWM) mask including white matter tissue with no lesions and anomalies from the ONDRI lesion mask of the subject, considering that in the ONDRI mask voxels with a grey-value of 1 are part of NAWM

command_16 = 'fslmaths ONDRI_lesion_mask_Subject_0.nii.gz.nii.gz -uthr 1 NAWM_mask_Subject_0.nii.gz.nii.gz';

status_16 = system(command_16);

%*******************************************************************************************

% #####Step 9: Generating the non-lesion part of the segmented WM tracts (multiplying tracts' binary masks by ONDRI NAWM mask)

% fmajor: Corpus callosum - forceps major

command_17 = 'fslmaths Subject_0/dpath/fmajor_PP_avg33_mni_bbr/path.pd_toanatorig_resized_threshold_50_bin.nii.gz -mul NAWM_mask_Subject_0.nii.gz.nii.gz Subject_0/dpath/fmajor_PP_avg33_mni_bbr/path.pd_toanatorig_resized_threshold_50_bin_NAWM.nii.gz';

status_17 = system(command_17);

%*******************************************************************************************

% #####Step 10: Calculating the mean DTI metrics in non-lesioned part of the segmented WM tracts
```

```
% fmajor: Corpus callosum - forceps major

% FA in non-lesion part of famjor

command_18 = 'fslmaths
Subject_0/dpath/fmajor_PP_avg33_mni_bbr/path.pd_toanatorig_resized_threshold_50_bin_NAWM.nii.gz -mul
Subject_0/dmri/dtifit_FA_toanatorig_resized.nii.gz
Subject_0/dpath/fmajor_PP_avg33_mni_bbr/FA_in_NAWM.nii.gz';

status_18 = system(command_18);

%Reading the FA map in non-lesion part (NAWM part) of fmajor

FA_in_fmajor_in_NAWM=niftiread('Subject_0/dpath/fmajor_PP_avg33_mni_bbr/FA_in_NAWM.nii.gz');

mean_FA_in_fmajor_in_NAWM=meaan(find(FA_in_fmajor_in_NAWM));    %mean FA in non-lesion part of fmajor

% MD in non-lesion part of famjor

command_19 = 'fslmaths
Subject_0/dpath/fmajor_PP_avg33_mni_bbr/path.pd_toanatorig_resized_threshold_50_bin_NAWM.nii.gz -mul
Subject_0/dmri/dtifit_MD_toanatorig_resized.nii.gz
Subject_0/dpath/fmajor_PP_avg33_mni_bbr/MD_in_NAWM.nii.gz';

status_19 = system(command_19);

%Reading the MD map in non-lesion part (NAWM part) of fmajor

MD_in_fmajor_in_NAWM=niftiread('Subject_0/dpath/fmajor_PP_avg33_mni_bbr/MD_in_NAWM.nii.gz');

mean_MD_in_fmajor_in_NAWM=meaan(find(MD_in_fmajor_in_NAWM))*1000;    %mean MD in non-lesion part of
fmajor

% AD in non-lesion part of famjor

command_20 = 'fslmaths
Subject_0/dpath/fmajor_PP_avg33_mni_bbr/path.pd_toanatorig_resized_threshold_50_bin_NAWM.nii.gz -mul
Subject_0/dmri/dtifit_AD_toanatorig_resized.nii.gz
Subject_0/dpath/fmajor_PP_avg33_mni_bbr/AD_in_NAWM.nii.gz';

status_20 = system(command_20);

%Reading the AD map in non-lesion part (NAWM part) of fmajor

AD_in_fmajor_in_NAWM=niftiread('Subject_0/dpath/fmajor_PP_avg33_mni_bbr/AD_in_NAWM.nii.gz');

mean_AD_in_fmajor_in_NAWM=meaan(find(AD_in_fmajor_in_NAWM))*1000;    %mean AD in non-lesion part of
fmajor

% RD in non-lesion part of famjor

command_21 = 'fslmaths
Subject_0/dpath/fmajor_PP_avg33_mni_bbr/path.pd_toanatorig_resized_threshold_50_bin_NAWM.nii.gz -mul
Subject_0/dmri/dtifit_RD_toanatorig_resized.nii.gz
Subject_0/dpath/fmajor_PP_avg33_mni_bbr/RD_in_NAWM.nii.gz';

status_21 = system(command_21);

%Reading the RD map in non-lesion part (NAWM part) of fmajor

RD_in_fmajor_in_NAWM=niftiread('Subject_0/dpath/fmajor_PP_avg33_mni_bbr/RD_in_NAWM.nii.gz');

mean_RD_in_fmajor_in_NAWM=meaan(find(RD_in_fmajor_in_NAWM))*1000;    %mean RD in non-lesion part of
fmajor

%********************************************END***********************************************
```

Supplementary References

1. Adams HP, Jr., Bendixen BH, Kappelle LJ, et al. Classification of subtype of acute ischemic stroke. Definitions for use in a multicenter clinical trial. TOAST. Trial of Org 10172 in Acute Stroke Treatment. *Stroke*. Jan 1993;24(1):35-41. doi:10.1161/01.str.24.1.35

1. Ortiz GA, L. Sacco R. National Institutes of Health Stroke Scale (NIHSS). *Wiley StatsRef: Statistics Reference Online.*

2. Vanswieten JC, Koudstaal PJ, Visser MC, Schouten HJA, Vangijn J. Interobserver Agreement for the Assessment of Handicap in Stroke Patients. *Stroke*. May 1988;19(5):604-607. doi:Doi 10.1161/01.Str.19.5.60427.

3. Nasreddine ZS, Phillips NA, Bedirian V, et al. The Montreal Cognitive Assessment, MoCA: a brief screening tool for mild cognitive impairment. *J Am Geriatr Soc.* Apr 2005;53(4):695-9. doi:10.1111/j.1532-5415.2005.53221.x
